# Deficiency of ATF3 facilitates both angiotensin II‐induced and spontaneously formed aortic aneurysm and dissection development by activating cGAS–STING pathway

**DOI:** 10.1002/ctm2.70147

**Published:** 2024-12-27

**Authors:** Yifan Du, Poyi Hu, Xiangchao Ding, Dashuai Wang, Jingjing Luo, Sheng Le, Lingyun Ren, Manhua Chen, Ping Ye, Jiahong Xia

**Affiliations:** ^1^ Department of Cardiovascular Surgery Union Hospital, Tongji Medical College, Huazhong University of Science and Technology Wuhan China; ^2^ Department of Thoracic Surgery Renmin Hospital of Wuhan University Wuhan China; ^3^ Department of Cardiovascular Surgery The First Affiliated Hospital of Zhengzhou University Henan Province China; ^4^ Department of Thoracic Surgery Zhongnan Hospital of Wuhan University Wuhan University Wuhan China; ^5^ Department of Anesthesiology Central Hospital of Wuhan, Tongji Medical College, Huazhong University of Science and Technology Wuhan China; ^6^ Department of Cardiology Central Hospital of Wuhan, Tongji Medical College, Huazhong University of Science and Technology Wuhan China

**Keywords:** aortic aneurysm and dissection, cGAS–STING pathway, DNA stability, micronuclei formation, P21

## Abstract

**Background:**

Sporadic aortic aneurysm and dissection (AAD) is a critical condition characterised by the progressive loss of vascular smooth muscle cells (VSMCs) and the breakdown of the extracellular matrix. However, the molecular mechanisms responsible for the phenotypic switch and loss of VSMCs in AAD are not fully understood.

**Methods and results:**

In this study, we employed a discovery‐driven, unbiased approach. This approach encourages us to explore the unknown functions of activating transcription factor 3 (ATF3) rather than merely confirming existing hypotheses, while no assumptions were made about ATF3 prior to the experiments. We ensured the unbiased nature of our assessment by conducting morphological evaluations with two independent observers in a blinded manner. We identified elevated expression of ATF3 in both human sporadic AAD tissues and mouse AAD models. VSMC‐specific ATF3 conditional knockout (Atf3 cKO) mice showed notable enlargement, dissection and rupture in both thoracic and abdominal aortic regions after exposure to Ang II. Interestingly, older Atf3 cKO mice exhibited spontaneous aortic dissections and senescence of the aortic wall. Mechanistically, ATF3 deficiency led to the degradation of P21 through ubiquitination. Impaired DNA repair in VSMCs resulted in micronuclei formation in the cytoplasm, activating the  cyclicGMP‐AMP synthase‐ stimulator of interferon genes (cGAS–STING) pathway and inducing VSMC phenotypic switching and apoptosis. Finally, both pharmacological complementation of P21 function and knockdown of STING expression alleviated ATF3 deficiency‐induced AAD.

**Conclusions:**

Our study indicates that ATF3 is essential for genomic DNA stability in VSMCs through the P21–cGAS–STING pathway, suggesting that enhancing ATF3 expression in VSMCs could help prevent sporadic AAD.

**Key points:**

- ATF3 deficiency led to degradation of P21 through ubiquitination, which abolished the G1 phase arrest.
- VSMCs had no time window to repair the damaged DNA, leading to generation of micronuclei in cytoplasm.
- Cytoplasmic micronuclei facilitating the activation of cGAS–STING pathway, thus inducing the phenotypic switch and apoptosis of VSMCs

## Graphical abstract

1. ATF3 deficiency led to degradation of P21 through ubiquitination, which abolished the G1 phase arrest.
2. VSMCs had no time window to repair the damaged DNA, leading to generation of micronuclei in cytoplasm.
3. Cytoplasmic micronuclei facilitating the activation of cGAS–STING pathway, thus inducing the phenotypic switch and apoptosis of VSMCs

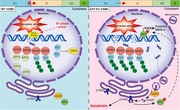

## 1 INTRODUCTION

Aortic aneurysm and dissection (AAD) is a common cardiovascular condition with significant mortality,^1^ and there are currently no effective treatments to prevent aortic degeneration or AAD progression. Aortic stress induces vascular smooth muscle cells (VSMCs) to transition from their primary contractile phenotype to diverse phenotypes, including proliferative, stressed and inflammatory states.^2^, ^3^, ^4^, ^5^, ^6^, ^7^, ^8^, ^9^, ^10^, ^11^ This transformation, a common characteristic of both thoracic and abdominal AAD, is associated with heightened activation and regulation of matrix metalloproteinases (MMPs), the ADAM family and the fibrinolytic pathway.^12^, ^13^

In the initial stages of AAD development, apoptosis leads to VSMC loss in the medial layer of the aortic wall. Numerous studies in humans and rodents have documented VSMC apoptosis in AAD tissue,^14^, ^15^, ^16^ with several mechanisms suggested for the transition of VSMCs to a more apoptosis‐prone senescent phenotype.^17^, ^18^ Therefore, addressing the phenotypic switch and VSMC loss due to apoptosis in the aortic wall could be a viable treatment strategy for AAD.

Oxidative stress‐induced DNA damage is prevalent in AAD, contributing to its development. From a pathophysiological perspective, mitochondrial DNA leakage activates the cGAS–STING pathway, leading to a phenotypic switch and loss of smooth muscle, which in turn decreases the stability of the aortic wall.^3^, ^24^ However, whether nuclear DNA damage influences VSMC homeostasis and AAD formation remains unknown.

By utilising single‐cell profiling, we identified the stress‐inducible transcription factor (TF) ATF3 as a novel regulator of VSMC phenotypic switching and apoptosis, through its role in maintaining genomic DNA stability in VSMCs. Our findings further demonstrate that ATF3 directly inhibits the ubiquitination and degradation of P21. By enhancing P21 stability, ATF3 promotes cell cycle arrest, allowing time for the repair of damaged VSMC DNA. ATF3 deletion in VSMCs impairs DNA repair, leading to micronuclei formation, which activates the cGAS–STING pathway, resulting in VSMC phenotypic switching and apoptosis. Overall, our results highlight the crucial role of ATF3 in regulating P21 activity, which is essential for maintaining genomic stability under genotoxic stress and inhibiting aortic degeneration. Thus, ATF3 is identified as a promising therapeutic target for AAD treatment.

## 2 MATERIALS AND METHODS

### 2.1 Human tissue study

Aortic tissue from the patient was obtained from the Department of Cardiovascular Surgery at Union Hospital, Tongji Medical College, Huazhong University of Science and Technology, Wuhan, China. Written informed consent was obtained from all patients and organ donors in compliance with the Declaration of Helsinki. Details of this section are provided in Supporting Information.

### 2.2 Animal studies

Mice were housed in a pathogen‐free facility at Tongji Medical College, Huazhong University of Science and Technology. A mouse model for sporadic AAD was developed by using ATF3 VSMC‐specific conditional knockout mice (C57BL/6J‐Atf3 fl/fl, Tagln Cre^+^) and their littermate controls (C57BL/6J‐Atf3 fl/fl, Tagln Cre^−^) which were constructed by Saiye Inc. (Suzhou, China). Additionally, ATF3 VSMC‐specific conditional knockout mice combined with Tmem173 knockdown (C57BL/6J‐Atf3 fl/fl, Tmem173 fl/+, Tagln Cre^+^) were generated by Model Organisms (Shanghai, China). Comprehensive procedural details can be found in Supporting Information.

In the angiotensin II (Ang II) infusion assay, mice received subcutaneous infusions of angiotensin II or saline via Alzet osmotic minipumps, which were implanted under 2% isoflurane anaesthesia.

In the porcine pancreatic elastase (PPE)‐induced AAA model, mice were anaesthetised with an intraperitoneal injection of 1% pentobarbital sodium and positioned supine on the control panel.

At the end of the experiments, animals were euthanised using CO_2_ or 2% isoflurane anaesthesia.

### 2.3 Statistical analysis

Representative figures and images are used to illustrate typical outcomes of each experiment. The Shapiro–Wilk test for normality and the Brown–Forsythe test for equality of group variance were performed on all data using Prism 9 software (GraphPad Software Inc., La Jolla, CA). Differences between two groups were evaluated using an unpaired, two‐tailed Student's *t*‐test for normally distributed data or a Mann–Whitney test for non‐normally distributed data. One‐way ANOVA was used to assess differences across groups for a single independent variable in multiple comparisons, while a two‐way ANOVA was used to examine differences across groups for two independent variables. *p* Values for pairwise comparisons were adjusted using the Bonferroni method when indicated. The figure legends specify the statistical tests used. Each experiment was conducted a minimum of three times. All data points represent independent samples, not technical replicates. Data are expressed as mean ± standard deviation.

## 3 RESULTS

### 3.1 ATF3 expression is elevated in both a mouse model of sporadic AAD and in human sporadic ATAAD tissues

We found that there was no significant immune cell infiltration in the aorta after 7 days of Ang II infusion, but there was a large amount of immune cell infiltration after 14 days (Figure S1). To assess the dynamic changes in aortic VSMCs during the early stages of AAD without the influence of immune cells, we performed single‐cell transcriptomic analysis on aortic tissues from C57 mice treated with either saline or Ang II (2000 ng/kg/min) for 7 days. Nineteen clusters were identified and categorised into VSMCs, endothelial cells, fibroblasts, mono/macrophages, T cells, B lymphocytes and neurons based on specific marker gene expression (Figures S2A and 1A). We extracted VSMC clusters for further analysis. We characterised VSMC subclusters by identifying differentially expressed genes (DEGs) for each cluster and conducting Gene Ontology (GO) analysis on these cluster‐specific DEGs to determine overrepresented functions (Figures S3, S4 and 1B). Clusters 4, 3 and 6 were identified as primary contractile VSMC clusters, demonstrating enhanced functions in contraction and extracellular matrix (ECM) organisation. Moreover, they were abundant in saline‐infused mice, indicating that they are unchallenged VSMCs. Cluster 9 was defined as ECM organisation VSMCs, which exhibited functions related to ECM organisation. Cluster 7 was characterised by contractile and ECM organisation attributes akin to clusters 3, 4 and 6, while also displaying stress‐related gene features such as *Nr4a1*, *Atf3*, *Klf2* and *Egr1*. Cluster 5 was defined as contractile/stressed/migration VSMCs, which showed enriched functions in response to mechanical stress, smooth muscle cell contraction and migration. Cluster 8 was defined as contractile/metabolically reprogrammed VSMCs, which exhibited features of contraction and increased glucose intake. Cluster 10 was characterised by inflammatory and pro‐apoptotic VSMCs, showing enhanced activity in inflammation and cell death processes. Cluster 1 was defined as stressed/proliferative VSMCs, which exhibited features of stress‐related genes and proliferative functions. Cluster 2 was defined as stressed/biogenesis VSMCs, which exhibited features of stress‐related genes and biogenesis functions.

**FIGURE 1:**
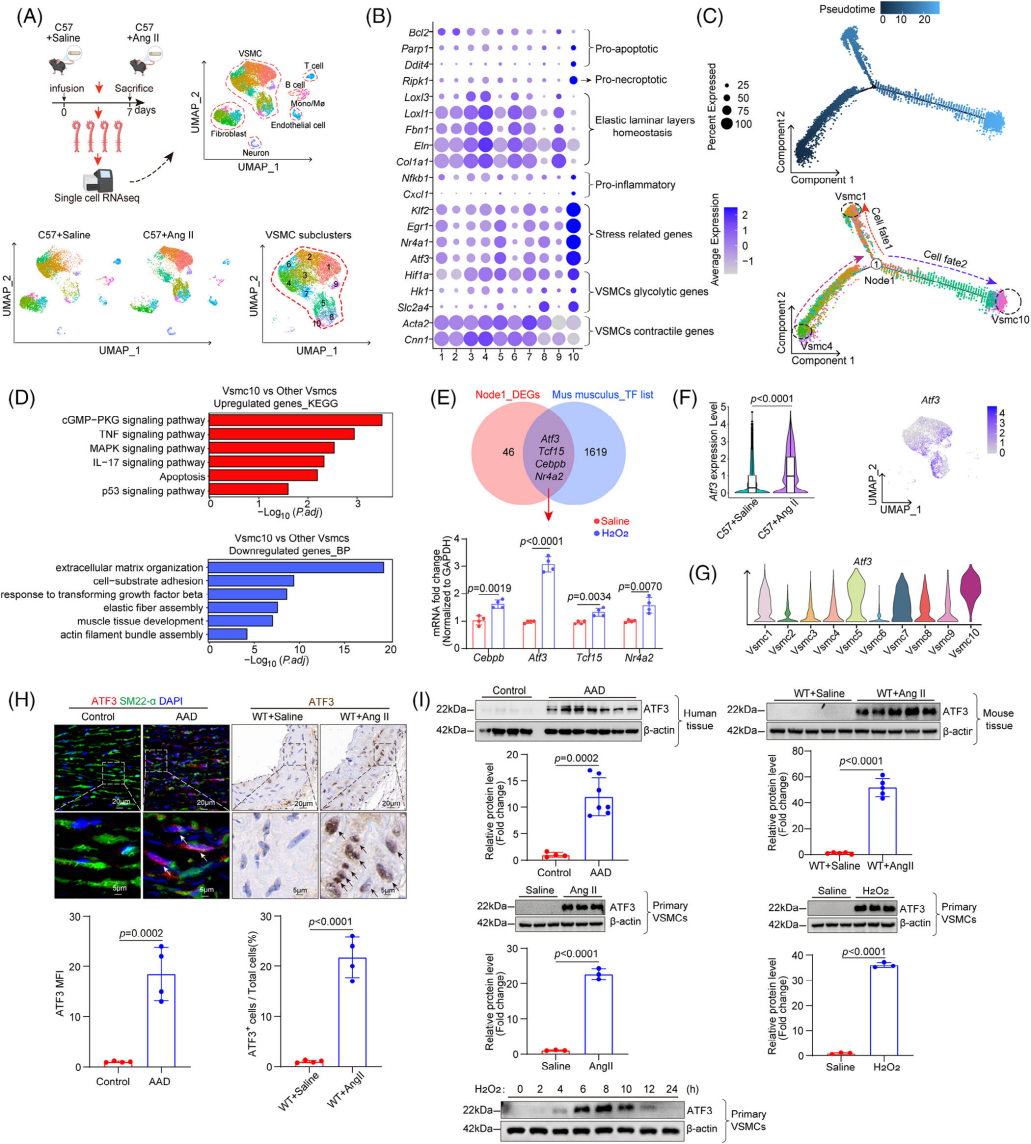
ATF3 Expression is consistently increased in mouse model of sporadic AAD and human sporadic ATAAD tissues. (A) Workflow for obtaining single‐cell RNA‐sequencing (scRNA‐seq) data and two‐dimensional uniform manifold approximation and projection (UMAP) plots. UMAP plot showing the subclusters of VSMCs. A total of 10 clusters were identified. (B) Dot plot showed the representative markers. (C) Pseudotime analysis was performed to estimate the transition of aortic VSMCs induced by aortic challenge. (D) Kyoto Encyclopedia of Genes and Genomes (KEGG) enrichment analysis of VSMC8 showing that aortic challenge led to different expression of several genes with roles in some biologic processes. (E) Four differentially expressed TF genes overlap between node 1 differentially expressed genes and mouse TF library and analysis of the mRNA levels of *Atf3*, *Tcf15*, *Nr4a2* and *Cebpb* after H_2_O_2_ stimulation (500 µM). (F) The expression level of *Atf3* in VSMCs from C57+Saline and C57+Ang II mice and The Featureplot of *Atf3*. (G) The violin plot showed the mRNA level of *Atf3* in VSMC subclusters. (H) Representative immunostaining and quantification data showing that the levels of ATF3 in aortic VSMCs were increased in AAD patients and in Ang II challenged wild type (WT) mice (*n* = 4 biological replicates per group, scale bar = 20 or 5 µm at different magnifications). (I) Up and middle panel, western blot analysis showing that the expression of ATF3 in the aorta was increased in AAD patients (*n* = 4 biological replicates in control group, *n* = 7 biological replicates in AAD group) and Ang II challenged mice (*n* = 5 biological replicates per group). Low panel, western blot analysis showing that the expression of ATF3 in H_2_O_2_ or Ang II challenged primary VSMCs was increased (*n* = 3 biological replicates per group). Its expression peaked 6 h, maintained for 2 h and then was down‐regulated. MFI, mean fluorescence intensity.

Pseudotime analysis using Monocle2 revealed two directions of VSMC transition: from contractile VSMC4 to inflammatory/pro‐death VSMC10 and from contractile VSMC4 to stressed/proliferative VSMC1 (Figures 1C,D and S5A,B). The VSMC10 cluster progressively down‐regulated contractile gene expression and up‐regulated genes associated with inflammation and apoptosis, suggesting it represents the terminal stage of VSMC differentiation following Ang II exposure. Additionally, we performed branch‐dependent expression analysis to identify DEGs driving VSMC differentiation (Figure S5A). The context‐specific regulation of gene expression is mainly achieved through TFs. Further analysis revealed that four differentially expressed TF genes, *Atf3*, *Tcf15*, *Nr4a1* and *Cebpb*, overlapped between node1 DEGs and the mouse TF library. Analysis of the mRNA levels of these four TFs after H_2_O_2_ stimulation in vitro showed that ATF3 exhibited the most significant changes (Figure 1E). Additionally, the mRNA level of ATF3 was significantly up‐regulated in the Ang II‐challenged Apoe−/− or C57 mouse aortas, especially in VSMCs (Figures 1F and S2B,C). Importantly, among all VSMC clusters, VSMC10 showed the highest expression of ATF3 (Figure 1G). Increased ATF3 levels significantly decreased in cells transitioning to VSMC1 but remained elevated in those transitioning to VSMC10, indicating ATF3 as a potential driver influencing VSMC fate (Figure S5A). To avoid the interference of immune cells, we selected adjacent tissue from the AAD lesion for detection. Consistent with the transcriptomic data, ATF3 protein levels were significantly increased in both the sporadic AAD mouse model and human sporadic AAD tissues (Figure 1H,I). In primary mouse VSMCs cultured in vitro, ATF3 was highly induced after stimulation with Ang II or H_2_O_2_. Its expression peaked at 6 h, was maintained for 2 h and then down‐regulated (Figure 1I). These findings indicate that ATF3 is significantly up‐regulated in AAD and may contribute to arterial remodelling.

### 3.2 VSMC‐specific ATF3 knockout aggravates AAD development

**FIGURE 2:**
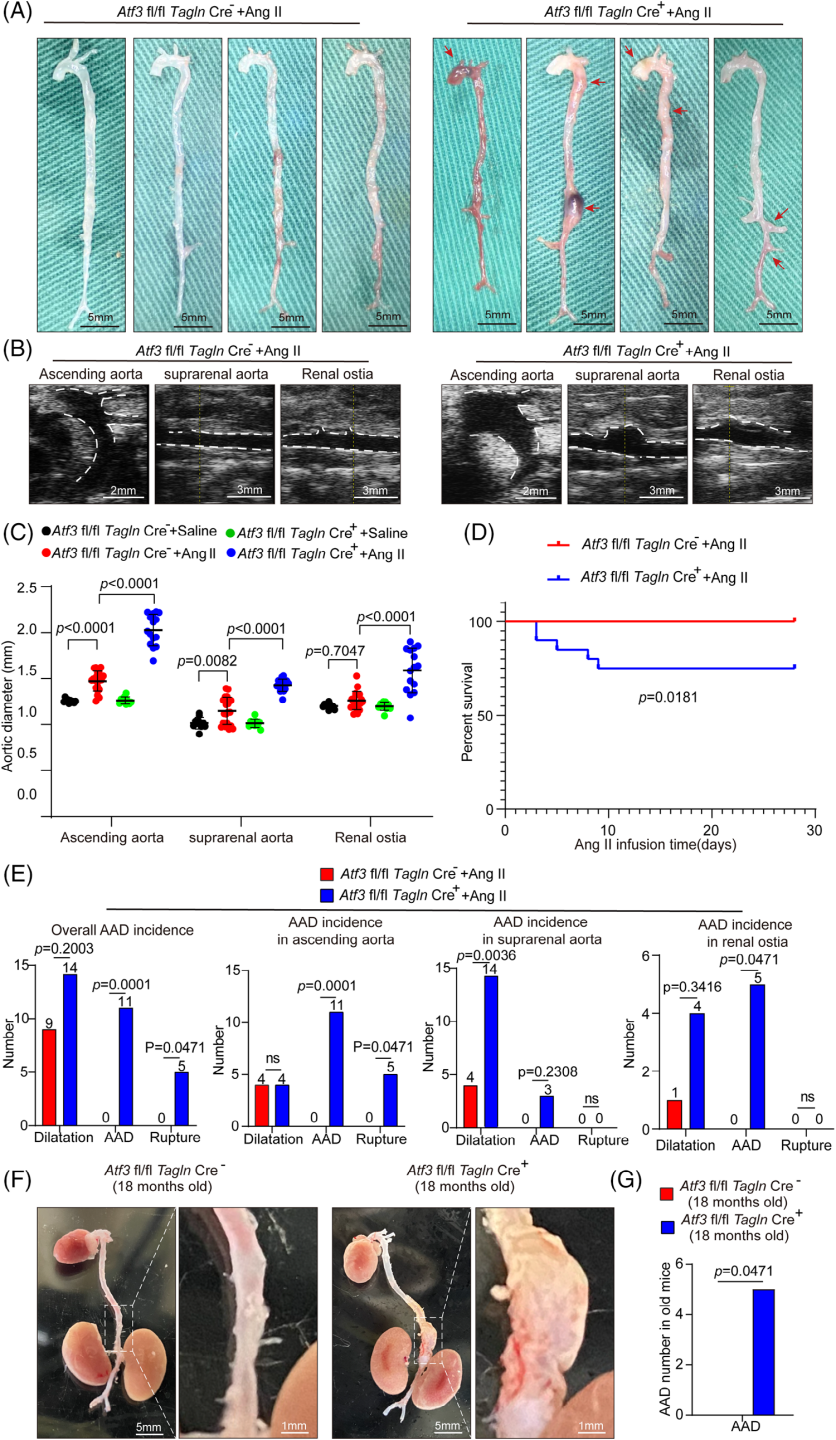
VSMCs‐specific ATF3 ablation aggravates Ang II challenged AAD development. *Atf3* cKO mice and littermate control mice were challenged with angiotensin II (Ang II) infusion (2000 ng/min/kg) for 4 weeks. (A) Representative images of excised aortas showing severe aortic enlargement and AAD formation in Ang II challenged *Atf3* cKO mice than in Ang II challenged littermate control mice (*n* = 20 biological replicates per group, scale bar = 5 mm). (B) Representative ultrasound images of different aortic segments after Ang II infusion for 28 days. White dash: ascending aortas or abdominal aortas (ascending aorta scale bar = 2 mm, abdominal aorta scale bar = 3 mm). (C) Mean aortic diameters of various aortic segments were larger in challenged *Atf3* cKO mice than in littermate control mice. The measurements were based on the excised aortas (*n* = 10 biological replicates in *Atf3* fl/fl *Tagln* Cre^−^+saline or *Atf3* fl/fl *Tagln* Cre^+^+saline group, *n* = 20 biological replicates in *Atf3* fl/fl *Tagln* Cre^−^+Ang II group, *n* = 15 biological replicates in *Atf3* fl/fl *Tagln* Cre^+^+Ang II group). (D) Kaplan–Meier survival analysis showing aggravated survival in challenged *Atf3* cKO mice compared with challenged littermate control mice during the 28 days of angiotensin II infusion. (E) The incidence of AAD in different aortic segments was significantly higher in challenged *Atf3* cKO mice than in littermate control mice. (F and G) Representative images of excised aortas showing spontaneous AAD formation in 18 months old *Atf3* cKO mice (*n* = 20 biological replicates per group, scale bar = 5 or 1 mm in different magnifications). The Fisher exact test was used for E and G, Two‐way ANOVA with the Bonferroni post hoc test was used for pairwise comparisons in C.

We created mice with a conditional ATF3 knockout in VSMCs (Atf3 cKO) to study the role of ATF3 in smooth muscle lesion formation. These mice underwent high‐dose Ang II infusion (2000 ng/min/kg) without being fed a high‐fat diet. Blood pressure was similarly increased in both the control and Atf3 cKO groups (Figure S6A). However, Atf3 cKO mice exhibited significant aortic degeneration and diameter enlargement upon challenge (Figure 2A–C). In addition, 25% of Atf3 cKO mice died (Figure 2D). Atf3 cKO mice exposed to Ang II showed an increased incidence of AAD (55%, *p* = .0001, encompassing both AAD) and rupture (25%, *p* = .0471), especially in the ascending aorta, suprarenal aorta and renal ostia (Figure 2E). Interestingly, without any intervention, five out of 20 aged Atf3 cKO mice (18 months old) exhibited spontaneous AAD formation (Figure 2F,G).

To further validate ATF3's involvement in AAD progression, we assessed aortic diameter differences between Atf3 cKO mice and their control littermates using a PPE‐induced abdominal aortic aneurysm (AAA) model, in which PPE degrades elastic fibres, causing aortic damage and aneurysm development. Our study demonstrated that the application of PPE to the infrarenal aorta resulted in significant aortic enlargement in Atf3 cKO mice compared with the littermate control group (Figure S6B). Results from repeated modelling (Figure S7) and modelling in female Atf3 cKO mice (Figure S8) also supported the previous conclusions. The absence of Atf3 significantly exacerbated AAD development, and no gender differences were observed in the role of ATF3 in AAD.

### 3.3 ATF3 deficiency aggravates aortic degeneration, VSMC phenotypic switch and apoptosis

**FIGURE 3:**
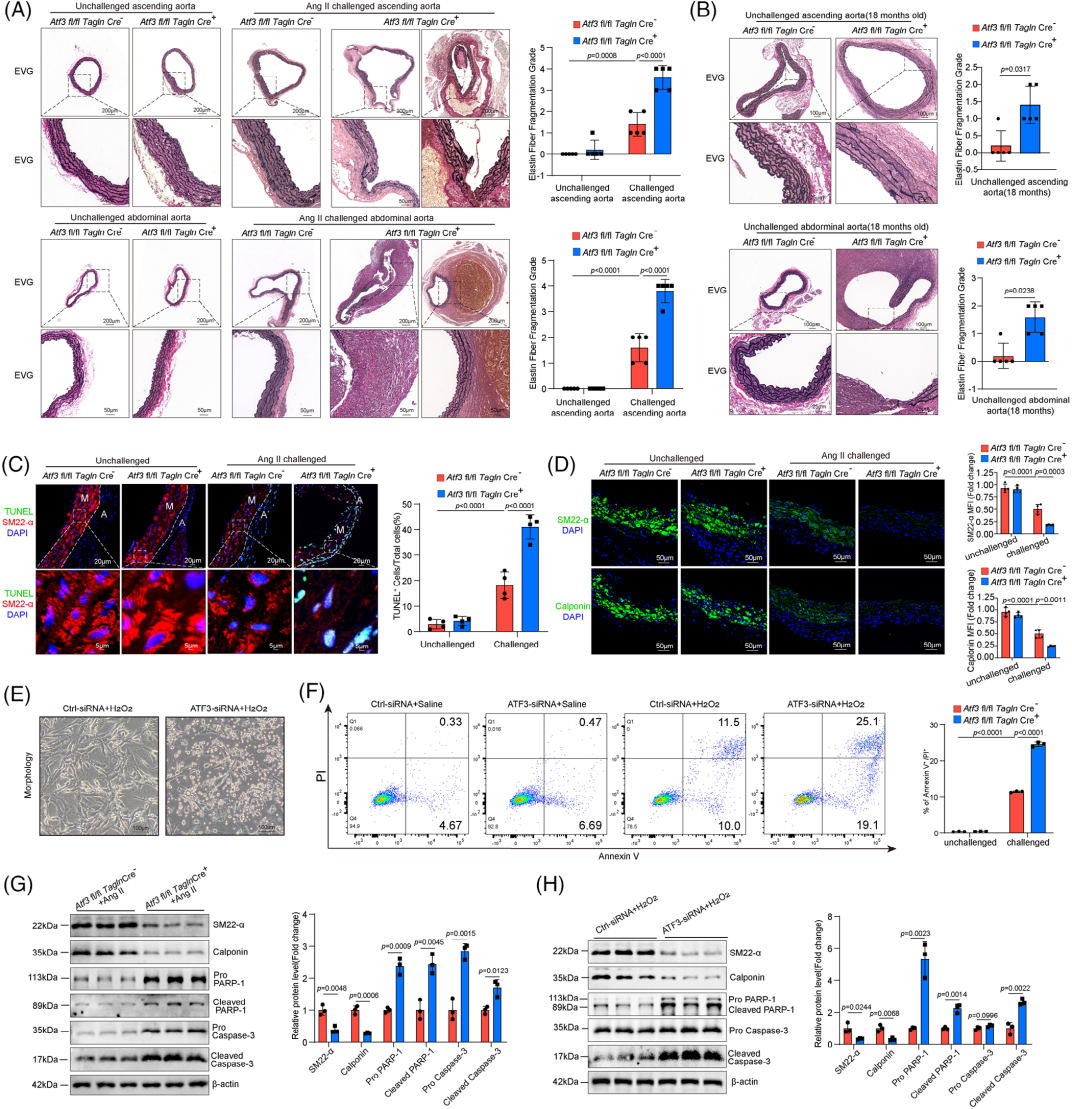
ATF3 deficiency aggravates aortic degeneration, VSMCs phenotypic switch and apoptosis. (A and B) Representative images of Elastica van Gieson (EVG) staining of different aortic segments in Ang II challenged mice and unchallenged old mice (*n* = 5 biological replicates per group, scale bar = 200 or 50 µm in A, scale bar = 100 or 25 µm in B). (C) Representative terminal deoxynucleotidyl transferase dUTP nick end labelling (TUNEL)‐stained images and quantification data showing that the number of apoptotic aortic VSMCs was increased in challenged Atf3 cKO mice compared with challenged littermate control mice (*n* = 4 biological replicates per group, scale bar = 20 or 5µm in different magnifications) (M: media; A: adventitia). (D) Representative immunofluorescence staining showing that the levels of SM22‐α and Calponin in aortic VSMCs were reduced in challenged Atf3 cKO mice compared with challenged littermate control mice (*n* = 4 biological replicates per group, scale bar = 50 µm). (E) The morphology of H_2_O_2_ challenged VSMCs with ATF3 knockdown (*n* = 3 biological replicates per group, scale bar = 100µm). (F) Flow cytometry analysis of H_2_O_2_ challenged VSMCs with ATF3 knockdown (*n* = 3 biological replicates per group). (G) Western blot analysis showing that the expression of Calponin and SM22‐α was decreased, Cleaved‐caspase3 and Cleaved‐PARP1 was increased in the aortas of challenged *Atf3* cKO mice (*n* = 3 biological replicates per group). (H) Western blot analysis showing that the expression of Calponin and SM22‐α was decreased, Cleaved‐caspase3 and Cleaved‐PARP1 was increased in H_2_O_2_ challenged VSMCs with ATF3 knockdown compared with H_2_O_2_ challenged VSMCs without ATF3 knockdown (*n* = 3 biological replicates per group). Unpaired two‐tailed *t*‐test was used in G and H. Mann–Whitney test (two‐sided) was performed for B. Two‐way ANOVA with the Bonferroni post hoc test was used for pairwise comparisons in A, C, D and F. MFI, mean fluorescence intensity.

Histologic analysis revealed that the aortas from Ang II‐challenged Atf3 cKO mice exhibited damaged aortic architecture, including a reduction in the VSMC layer and increased elastic fibre fragmentation, compared with aortas from littermate control mice (Figure 3A). In aged mice, Atf3 cKO mice showed thinner elastic lamellae and more severe elastic fibre fragmentation (Figure 3B). In Atf3 cKO mice, aortic challenge led to an increased number of  terminal deoxynucleotidyl transferase‐mediated dUTP nick end labeling (TUNEL)‐positive VSMCs in the aortic lesion and decreased expression of contractile proteins (Figure 3C,D). Consistently, in vitro experiments showed that knocking down ATF3 led to a significant increase in apoptosis when exposed to oxidative stress (Figure 3E,F). Western blot analysis of aortas from Atf3 cKO mice revealed significantly elevated levels of cleaved Caspase‐3 and cleaved poly ADP‐ ribose polymerase (PARP)‐1 (Figure 3G). In vitro experiments further demonstrated that silencing ATF3 in VSMCs resulted in decreased expression of VSMC contractile genes and increased levels of cleaved Caspase‐3 and cleaved PARP‐1 (Figure 3H). In vivo experiments revealed that ATF3 deficiency notably elevated osteopontin(OPN) expression in VSMCs following Ang II exposure, whereas in vitro experiments showed no significant change (Figure S9A–C). Meanwhile, the deficiency of ATF3 aggravated the production of matrix metalloproteinase (MMP)‐9, whereas MMP‐2 showed no significant difference in Ang II challenged VSMCs (Figure S9D). These results collectively suggest that the up‐regulation of ATF3 during AAD development acts as a self‐protective mechanism against arterial remodelling.

### 3.4 ATF3 negatively regulates the cGAS–STING pathway in VSMCs

**FIGURE 4:**
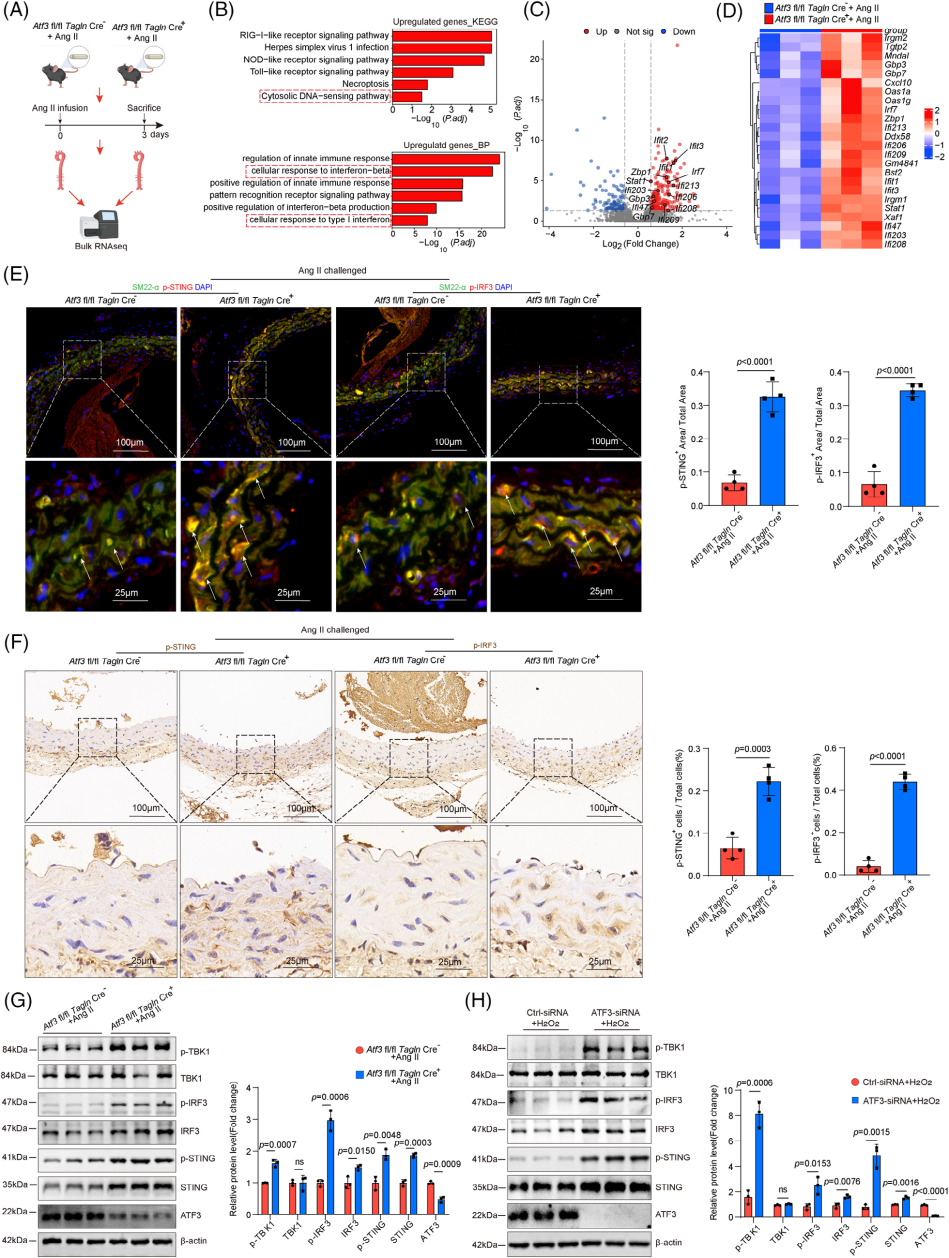
Loss of ATF3 in VSMCs leads to the activation of cGAS–STING pathway. (A) Bulk RNA‐sequencing (RNA‐seq) analysis was performed in aortas from Atf3 cKO mice and littermate control mice challenged with Ang II infusion for 3 days. For each group, two aortas were pooled as one sample, and duplicate samples were tested (*n* = 3 biological replicates per group). (B) Kyoto Encyclopedia of Genes and Genomes (KEGG) analysis and Gene Ontology (GO) analysis showing that aortic challenge induced the expression of genes involved in several biologic processes related to cGAS–STING pathway. (C and D) Volcano plot and Heatmap showing that cGAS–STING pathway related genes were up‐regulated in Ang II challenged *Atf3* cKO mice compared with challenged littermate control mice. (E) Representative immunofluorescence staining and quantification data showing that the levels of p‐STING and p‐IRF3 in aortic VSMCs were increased in challenged *Atf3* cKO mice compared with challenged littermate control mice (*n* = 4 biological replicates per group, scale bar = 100 or 25 µm in different magnifications). (F) Representative immunohistochemistry staining and quantification data showing that the levels of p‐STING and p‐IRF3 in aortic VSMCs were increased in challenged *Atf3* cKO mice compared with challenged littermate control mice (*n* = 4 biological replicates per group, scale bar = 100 or 25 µm in different magnifications). (G) Western blot analysis showing that the expression of p‐STING, STING, p‐IRF3, IRF3 and p‐TBK1 were increased in Angiotensin II challenged *Atf3* cKO mice aortas compared with challenged littermate control mice (*n* = 3 biological replicates per group). (H) Western blot analysis showing that the expression of p‐STING, STING, p‐IRF3, IRF3 and p‐TBK1 were increased in H_2_O_2_ challenged VSMCs with ATF3 knockdown compared with VSMCs without ATF3 knockdown (*n* = 3 biological replicates per group). Unpaired two‐tailed *t*‐test was used in E, F, G and H. MFI, mean fluorescence intensity.

**FIGURE 5:**
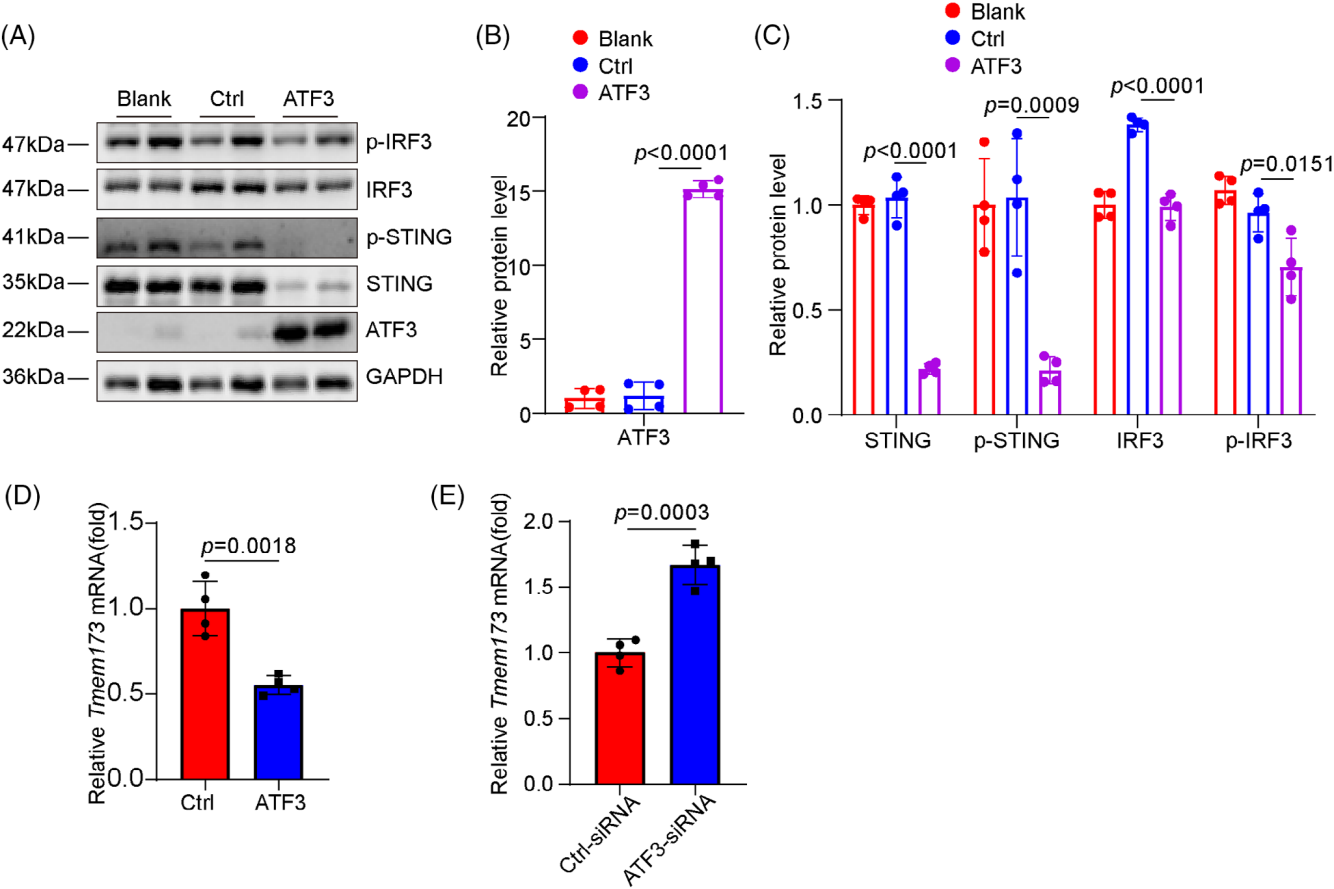
Overexpression ATF3 suppressed cGAS–STING pathway activation. (A–C) Overexpression of ATF3 in VSMCs down‐regulated the level of STING, p‐STING, IRF3 and p‐IRF3 (*n* = 4 biological replicates per group). (D) Overexpression of ATF3 in VSMCs induced *Tmem173* mRNA level (*n* = 4 biological replicates per group). (E) ATF3 knockdown enhanced *Tmem173* mRNA level (*n* = 4 biological replicates per group). One‐way ANOVA with the Bonferroni post‐hoc test for pairwise comparisons was used for B and C. Unpaired two‐tailed *t*‐test was used in D and E.

We explored the mechanisms through which ATF3 regulates VSMC homeostasis. We performed bulk RNA sequencing on aortas from Atf3 cKO and littermate control mice 3 days post‐Ang II infusion to examine the initial phase of the aortic stress response (Figure 4A). GO and Kyoto Encyclopedia of Genes and Genomes (KEGG) analyses revealed that aortic challenge in Atf3 cKO mice led to increased expression of genes associated with biological processes such as the inflammatory response, cytosolic DNA‐sensing pathway and cellular response to type I interferon, indicating activation of the cGAS–STING pathway (Figure 4B–D). To avoid the influence of immune cells, descending aortas were collected for immunostaining of the cGAS–STING pathway. Our transcriptomic study showed that aortic challenge in Atf3 cKO mice led to a significant increase in the protein levels of phospho‐interferon regulatory factor 3 (p‐IRF3), p‐STING and phospho‐TANK‐ binding kinase 1 (p‐TBK1) in the aortic wall (Figure 4E–G), and elevated interferon (IFN)‐β levels in serum (Figure S10). Additionally, in vitro experiments demonstrated that after 6 h of 500 µM H_2_O_2_ stimulation, cultured primary mouse VSMCs with silenced Atf3 exhibited significantly higher levels of p‐STING, p‐IRF3 and p‐TBK1 compared with controls (Figure 4H). Meanwhile, as a non‐canonical downstream molecule of STING, p65 was more significantly activated in ATF3 knockout VSMCs compared with the control group (Figure S11A). Overexpression of ATF3 suppressed the cGAS–STING pathway (Figures 5A–C and S12). Moreover, ATF3 also directly regulated STING mRNA levels (Figure 5D,E). Taken together, these results support the notion that ATF3 negatively regulates the cGAS–STING pathway in VSMCs.

### 3.5 Loss of ATF3 leads to severe DNA damage and increased micronuclei formation

**FIGURE 6:**
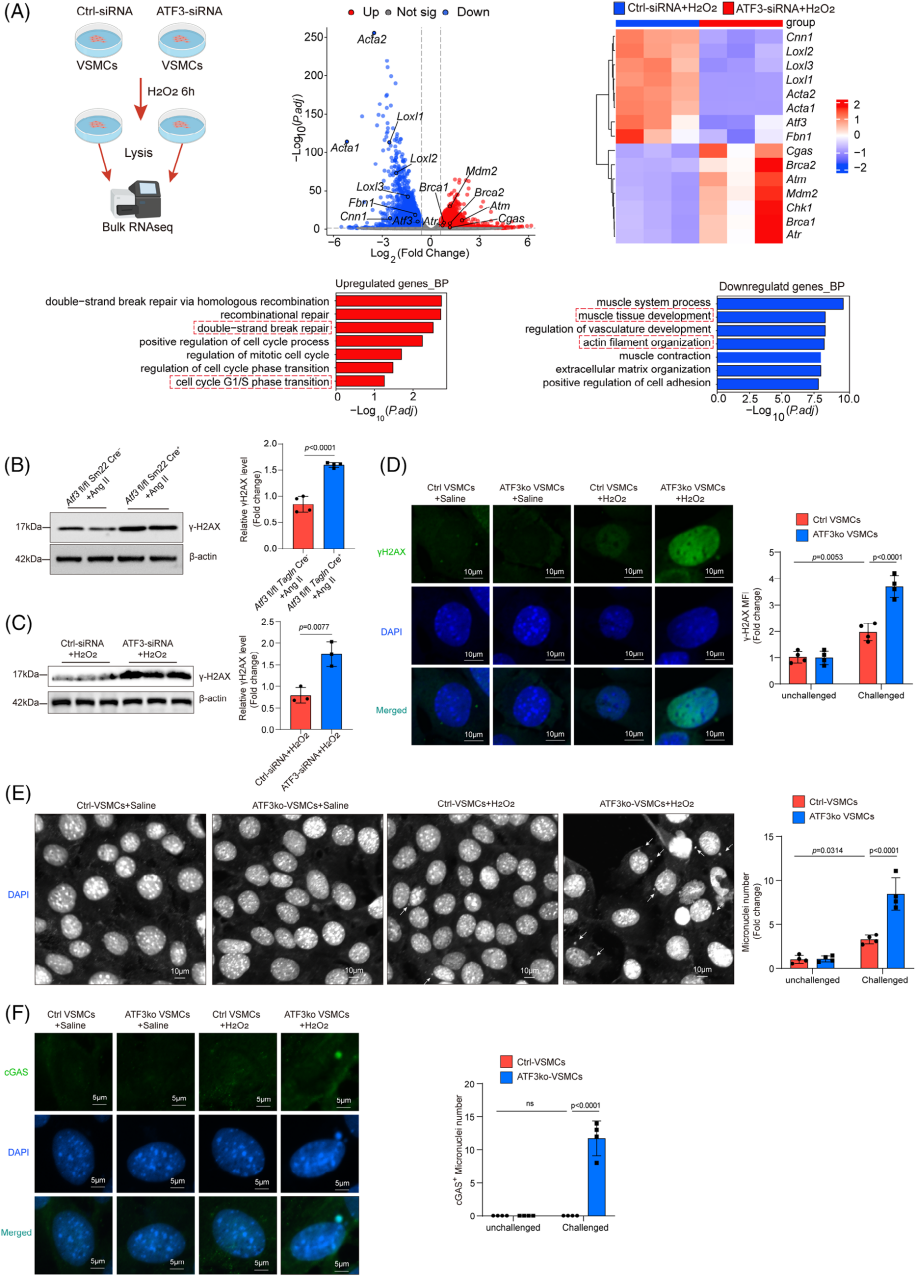
Loss of ATF3 leading to more severe DNA damage and facilitating micronuclei formation. In vitro experiments, the VSMCs were challenged for 6 h. A, Bulk RNA‐sequencing (RNA‐seq) analysis was performed in H_2_O_2_ challenged VSMCs with *Atf3* knockdown (*n* = 3 biological replicates per group). Volcano plot and Heatmap showing different gene expression patterns among groups. Volcano plot, Heatmap and GO analysis showing that aortic challenge induced the expression of genes involved in DNA repair. (B) Western blot analysis and quantification data showing that the expression of γ‐H2AX was increased in the aorta of challenged *Atf3* cKO mice (*n* = 4 biological replicates per group). (C) Western blot analysis and quantification data showing that the expression of γ‐H2AX was increased in VSMCs with ATF3 knockdown (*n* = 3 biological replicates per group). (D) Representative immunofluorescence staining and quantification data showing that the levels of γ‐H2AX were increased in cultivated aortic VSMCs from *Atf3* cKO mice after H_2_O_2_ challenge compared with other groups (*n* = 4 biological replicates per group, scale bar = 10 µm). (E) Confocal immunofluorescence analysis and quantification data showing that ATF3 deficiency promoted the formation of micronuclei (*n* = 4 biological replicates per group, scale bar = 10 µm). (F) Confocal immunofluorescence analysis and quantification data showing that ATF3 deficiency increased the co‐localisation of cGAS and micronuclei (*n* = 4 biological replicates per group, scale bar = 5 µm). Unpaired two‐tailed *t*‐test was used in B and C. Two‐way ANOVA with the Bonferroni post hoc test was used for pairwise comparisons in D, E and F. MFI, mean fluorescence intensity.

To further investigate the mechanism through which Atf3 knockout promotes cGAS–STING pathway activation, cultured primary VSMCs with or without Atf3 knockdown were subjected to bulk RNA sequencing after 6 h of stimulation with 500 µM H_2_O_2_. GO enrichment analysis revealed the activation of DNA double‐strand break repair‐related pathways and cell cycle phase transition pathways in Atf3 knockdown cells (Figure 6A). Consistent with the sequencing results, the level of γ‐H2AX was up‐regulated in aortic tissue of challenged Atf3 cKO mice as well as in H_2_O_2_‐treated VSMCs with ATF3 knockdown, indicating that Atf3 insufficiency aggravates genomic DNA damage (Figure 6B,C). In addition, these results were further demonstrated through immunofluorescence staining in ATF3‐knockout primary VSMCs (Figure 6D). Meanwhile, Atf3 insufficiency facilitated the production of micronuclei in challenged VSMCs (Figure 6E). Furthermore, confocal immunofluorescence showed that cGAS co‐localised with micronuclei in challenged ATF3‐knockout primary VSMCs (Figure 6F), revealing increased recruitment of cGAS to micronuclei. These findings suggest that ATF3 plays a critical role in maintaining genomic DNA stability.

### 3.6 ATF3 maintains the stability of P21

**FIGURE 7:**
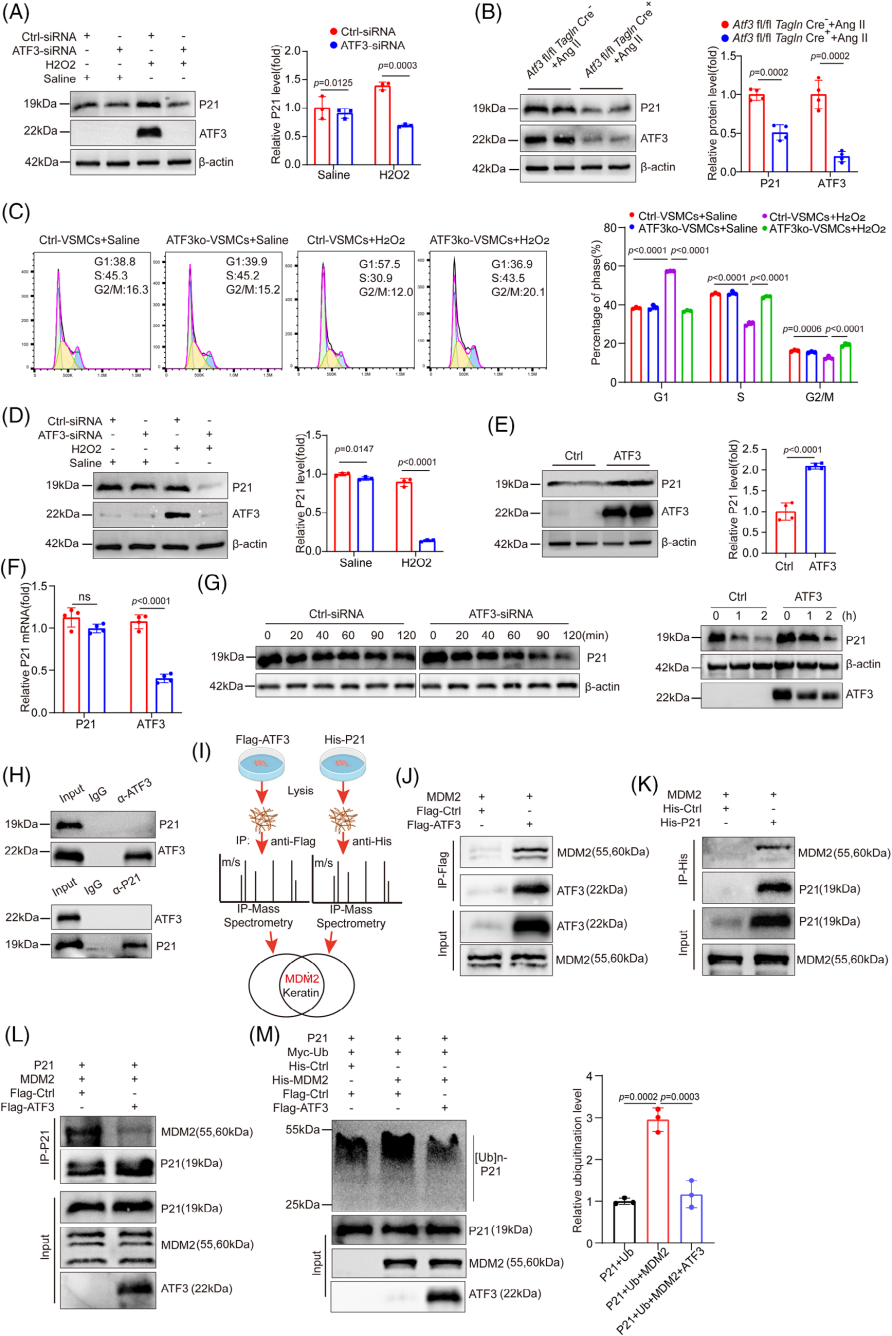
ATF3 contributes to maintaining the stability of P21. (A) Western blot analysis and quantification data showing that the expression of P21 was decreased in H_2_O_2_ challenged VSMCs with ATF3 knockdown (*n* = 3 biological replicates per group). (B) Western blot analysis and quantification data showing that the expression of P21 was decreased in the aorta of challenged Atf3 cKO mice (*n* = 4 biological replicates per group). (C) Flow cytometry analysis revealed that ATF3 deficiency abolished cell cycle arrest in the G1 phase and facilitated the entrance to S and G2/M phase (*n* = 3 biological replicates per group). (D) Western blot analysis and quantification data showing that the expression of P21 was decreased in H_2_O_2_ challenged P53‐null H1299 cells with ATF3 knockdown (*n* = 3 biological replicates per group). (E) Western blot analysis and quantification data showing that the expression of P21 was increased P53‐null H1299 cells transfected with ATF3 (*n* = 4 biological replicates per group). (F) Knockdown of ATF3 expression decreased the protein level but not the mRNA level of P21. VSMCs expressing ATF3‐siRNA or Ctrl‐siRNA were treated with 500 µM H_2_O_2_ for 6 h and then were tested (*n* = 4 biological replicates per group). (G) ATF3 knockdown decreased the P21 stability. VSMCs expressing ATF3‐siRNA or Ctrl‐siRNA were treated with 100 µg/mL of cycloheximide (CHX) and lysed for western blotting as indicated. ATF3 overexpression increased the P21 stability. VSMCs were transfected with ATF3 or Ctrl plasmid for 2 days and then were treated with 100 µg/mL of cycloheximide (CHX) and lysed for western blotting as indicated. (H) Reciprocal co‐IP assays showing that ATF3 and P21 had no direct protein–protein interaction. 293T cells in 60‐mm dishes were co‐transfected with 3 µg of p21 or 3 µg of ATF3 and subjected to co‐IP using 1 µg of the ATF3 or the P21 antibody. (I) Procedure of identifying the overlapped Proteins interacting with ATF3 and P21. (J) The interaction between the ATF3 and MDM2 proteins was confirmed by co‐IP assays. 293T cells expressing a FLAG‐tagged ATF3 and MDM2 protein were subjected to co‐IP using the FLAG antibody. (K) The interaction between the P21 and MDM2 proteins was confirmed by co‐IP assays. 293T cells expressing a His‐tagged P21 and MDM2 protein were subjected to co‐IP using the His antibody. (L) ATF3 expression decreased the interaction between P21 and MDM2. Lysates from 293T cells transfected with 1 µg of P21, MDM2, Flag‐Ctrl/Flag‐ATF3 were subjected to immunoprecipitation using the P21 antibody followed by SDS‐PAGE. M, ATF3 expression decreased the P21 ubiquitination level. Lysates from 293T cells transfected with 0.6 µg of P21, Myc‐ubiquitin, His‐Ctrl/His‐MDM2, Flag‐Ctrl/Flag‐ATF3 were subjected to immunoprecipitation using the P21 antibody followed by SDS‐PAGE. Ubiquitinated proteins were detected by western blotting using the Myc antibody. Unpaired two‐tailed *t*‐test was used in B, E and F. Two‐way ANOVA with the Bonferroni post hoc test was used for pairwise comparisons in A, C and D.

Next, the effects of ATF3 on DNA double‐strand break repair‐related pathways and cell cycle phase transition pathways, particularly the G1/S phase transition, were investigated (Figure 6A). P21, the key regulator of the transition from G1 phase to S phase, was examined after ATF3 knockdown in cultured primary VSMCs. ATF3 knockdown significantly down‐regulated P21 (Figure 7A). Similarly, aortic tissues from challenged Atf3 cKO mice showed decreased P21 levels (Figure 7B). Flow cytometric analysis indicated that ATF3 deficiency eliminated G1 phase cell cycle arrest, promoting progression to the S and G2/M phases (Figure 7C). To further evaluate the effects of ATF3 on P21 expression, both loss‐ and gain‐of‐function approaches were carried out in P53‐null H1299 cells. Importantly, the expression pattern of P21 aligned with that of ATF3, indicating that ATF3 regulates P21 in a P53‐independent manner (Figure 7D,E). These effects are unlikely to be attributed to the inhibition of P21 transcription, as ATF3 siRNA did not reduce P21 mRNA levels (Figure 7F), suggesting post‐transcriptional regulation of P21. Consistent with our hypothesis, ATF3 was found to increase the half‐life of endogenous P21 protein, while the loss of ATF3 led to increased degradation of P21 (Figure 7G). In summary, the findings indicate that ATF3 enhances P21 protein stability.

We then investigated whether ATF3 binds to P21 and affects its stability. Reciprocal co‐immunoprecipitation (co‐IP) experiments indicated that ATF3 and P21 were absent from the same immune complex (Figure 7H), implying that ATF3 does not directly interact with P21. We hypothesised that ATF3's effect on P21 degradation may be mediated by other proteins. We utilised IP‐MS to identify proteins interacting with ATF3 and P21 to test this hypothesis. Among these overlapping proteins, we identified MDM2 as a potential intermediary molecule (Figure 7I). Additional co‐IP experiments demonstrated that both ATF3 and P21 could bind to murine double minute 2 (MDM2) (Figure 7J,K). Furthermore, ATF3 was found to reduce the binding of MDM2 to P21, as well as decreasing the ubiquitination of P21 by MDM2 (Figure 7L,M). The same conclusion was validated in VSMCs after H_2_O_2_ stimulation (Figure S13). Knockdown of MDM2 in ATF3‐knockout primary VSMCs elevated P21 protein levels, reduced DNA damage and decreased cGAS–STING pathway activation, suggesting MDM2's role in enhancing P21 degradation, promoting DNA damage and activating the cGAS–STING pathway in the absence of ATF3 (Figure S14). These findings suggest that ATF3 may stabilise P21 by reducing MDM2‐mediated ubiquitination.

### 3.7 Roscovitine treatment mitigates the adverse effects of ATF3 deficiency on VSMCs

**FIGURE 8:**
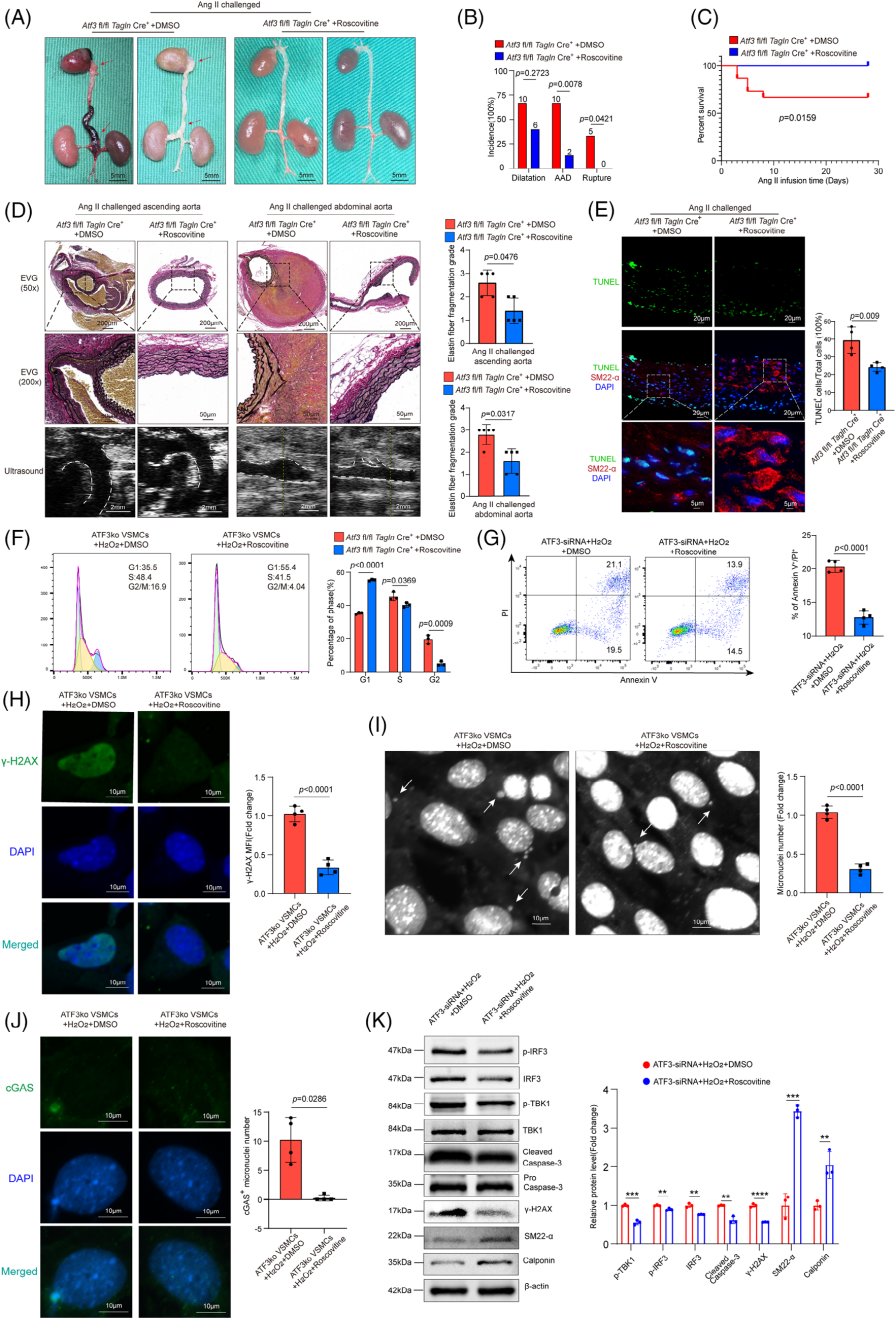
Roscovitine treatment mitigates the adverse effects of ATF3 ablation on VSMCs. Atf3 cKO mice were challenged with angiotensin II infusion (2000 ng/min/kg) and administrated with DMSO or roscovitine for 4 weeks. (A) Representative images of excised aortas showing mitigated aortic degeneration in *Atf3 cKO* mice administrated with roscovitine compared with *Atf3 cKO* mice administrated with DMSO (*n* = 15 biological replicates per group, scale bar = 5 mm). (B) The incidences of AAD and rupture were significantly decreased in *Atf3 cKO* mice administrated with roscovitine compared with *Atf3 cKO* mice administrated with DMSO. (C) Kaplan–Meier survival analysis showing decreased survival percentage in *Atf3 cKO* mice administrated with roscovitine compared with *Atf3 cKO* mice administrated with DMSO during the 4 weeks of angiotensin II infusion. the differences were analysed by using the log‐rank (Mantel–Cox) test. (D) Representative images of Elastica van Gieson (EVG) staining and ultrasound images of different aortic segments after Ang II infusion for 28 days. White dash: ascending aortas or abdominal aortas. *Atf3* cKO mice treated with roscovitine showed better preserved aortic structure, less aortic enlargement compared with the control‐treated mice (*n* = 5 biological replicates per group, scale bar = 200 or 50µm in EVG staining in different magnifications, scale bar = 2 mm in ultrasound images). (E) Representative terminal deoxynucleotidyl transferase dUTP nick end labelling (TUNEL)‐stained images of *Atf3 cKO* mice administrated with roscovitine or DMSO. Quantification data showing that the number of apoptotic aortic VSMCs was reduced in *Atf3 cKO* mice administrated with roscovitine compared with the control‐treated mice (*n* = 4 biological replicates per group, scale bar = 20 or 5 µm) (M: media; A: adventitia). (F) Roscovitine restored the function of P21, leading to G1 phase arrest (*n* = 3 biological replicates per group). (G) roscovitine partially alleviated H_2_O_2_‐induced VSMCs apoptosis (*n* = 4 biological replicates per group). (H) Roscovitine partially alleviated H_2_O_2_‐induced γ‐H2AX protein level elevation (*n* = 4 biological replicates per group, scale bar = 10 µm). (I and J) Roscovitine mitigated the formation of micronuclei and their co‐localisation with cGAS (*n* = 4 biological replicates per group, scale bar = 10µm). (K) Western blotting showed that roscovitine partially prevented the effects of ATF3 deficiency, including IRF3 and TBK1 phosphorylation, DNA damage, caspase‐3 cleavage, decrease of contractile proteins (SM22‐α and Calponin) (*n* = 3 biological replicates per group). The Fisher exact test was used for B. Unpaired two‐tailed *t*‐test was used in E–K. Mann–Whitney test (two‐sided) was performed for D. *p < .05, **p < .01, ***p < .001. ****p < .0001. MFI, mean fluorescence intensity.

After confirming the significance of the ATF3–P21–cGAS–STING axis in AAD development, we explored the potential of pharmacologically restoring P21 function to prevent aortic degeneration and AAD progression in mice. We investigated the impact of roscovitine, a reversible CDK2/4 inhibitor, on mitigating AAD development and progression in a mouse model of sporadic AAD. Atf3 cKO mice were challenged with Ang II infusion and also received either roscovitine (50 mg/kg) or corn oil (control) during Ang II infusion. Atf3 cKO mice treated with roscovitine exhibited superior aortic structure preservation, reduced aortic enlargement, significantly lower AAD incidence (13.3%, *p* = .0078) and rupture (0%, *p* = .0421), and enhanced survival rates compared with control‐treated mice (Figure 8A–C). In Ang II‐challenged Atf3 cKO mice, roscovitine treatment resulted in better preservation of elastic fibre architecture in the aortas compared with those without the treatment (Figure 8D). Furthermore, roscovitine treatment in Atf3 cKO mice led to decreased STING and IRF3 phosphorylation (Figure S16A,B) and a reduction in TUNEL‐positive cells in the aortic wall (Figure 8E). Conversely, roscovitine treatment restored the reduced contractile proteins SM22‐α and Calponin in the aortic wall of Atf3 cKO mice (Figure S16E,F).

In challenged VSMCs with ATF3 knockout, roscovitine restored the function of P21, leading to G1 phase arrest (Figure 8F). Meanwhile, roscovitine partially alleviated H_2_O_2_‐induced VSMC apoptosis and γ‐H2AX protein level elevation (Figure 8G,H). Moreover, roscovitine mitigated the formation of micronuclei and their co‐localisation with cGAS (Figure 8I,J). Western blotting showed that roscovitine partially prevented the effects of ATF3 deficiency, including IRF3 and TBK1 phosphorylation, DNA damage, caspase‐3 cleavage and the decrease in contractile proteins (SM22‐α and Calponin) (Figure 8K).

### 3.8 STING knockdown mitigates the adverse effects of ATF3 loss on VSMCs

**FIGURE 9:**
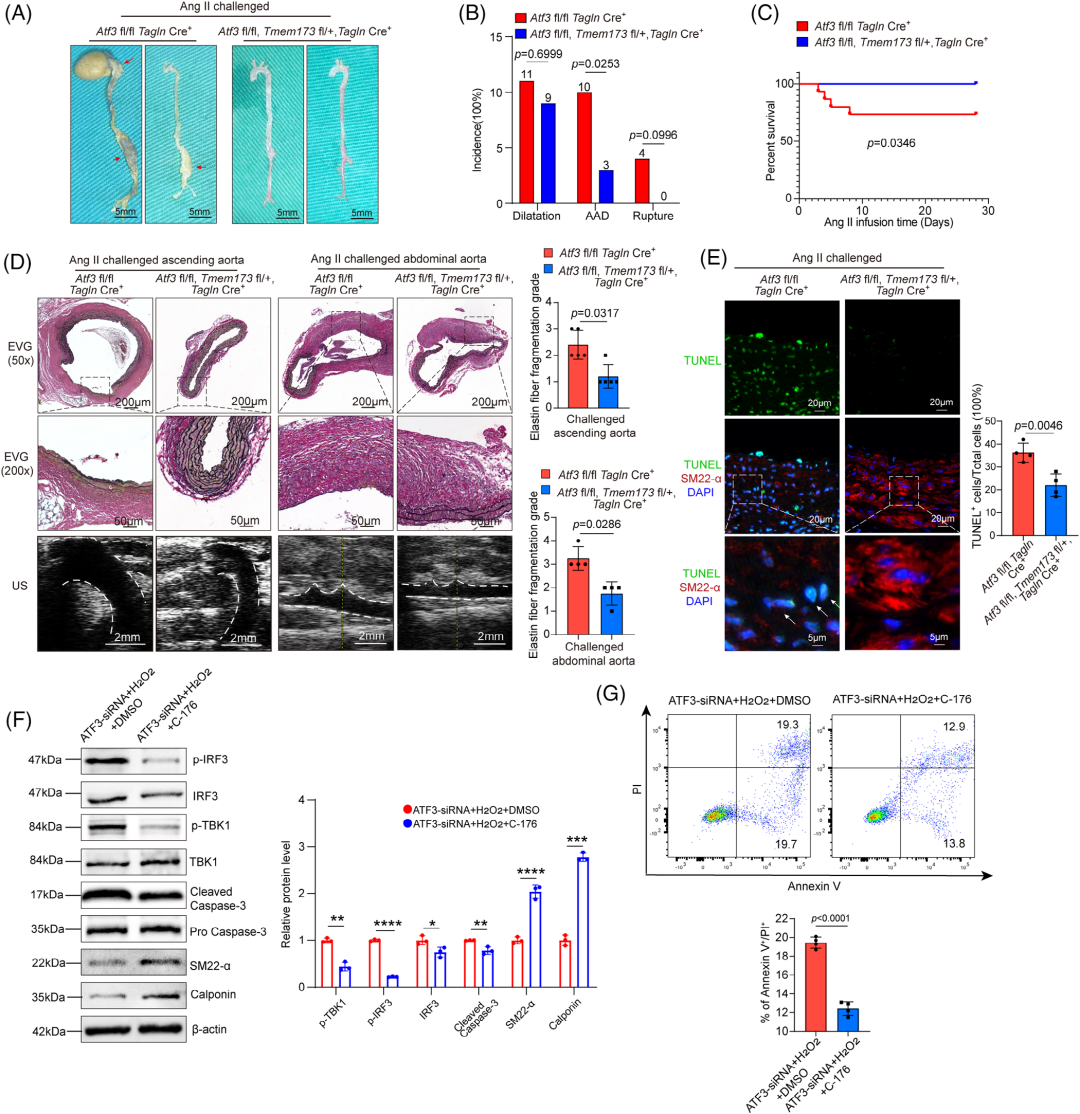
STING knockdown mitigates the adverse effects of ATF3 silencing on VSMCs. *Atf3* ^fl/fl^; *Tmem173* ^fl/+^; *Tagln*‐Cre^+^ mice *Atf3* ^fl/fl^ *Tagln*‐Cre^+^ mice were challenged with angiotensin II infusion (2000 ng/min/kg). (A) Representative images of excised aortas showing mitigated aortic degeneration in *Atf3* ^fl/fl^; *Tmem173* ^fl/+^; *Tagln*‐Cre^+^ mice compared with *Atf3* ^fl/fl^ *Tagln*‐Cre^+^ mice (*n* = 15 per group, scale bar = 5 mm). (B) The incidences of AAD and rupture were significantly decreased in *Atf3* ^fl/fl^; *Tmem173* ^fl/+^; *Tagln*‐Cre^+^ mice compared with *Atf3* ^fl/fl^ *Tagln*‐Cre^+^ mice. (C) Kaplan–Meier survival analysis showing decreased survival percentage in *Atf3* ^fl/fl^; *Tmem173* ^fl/+^; *Tagln*‐Cre^+^ mice compared with *Atf3* ^fl/fl^ *Tagln*‐Cre^+^ mice during the 4 weeks of angiotensin II infusion. the differences were analysed by using the log‐rank (Mantel–Cox) test. (D) Representative images of Elastica van Gieson (EVG) staining and ultrasound images of different aortic segments after Ang II infusion for 28 days. White dash: ascending aortas or abdominal aortas. *Atf3* ^fl/fl^; *Tmem173* ^fl/+^; *Tagln*‐Cre^+^ mice showed better preserved aortic structure, less aortic enlargement compared with *Atf3* ^fl/fl^; *Tagln*‐Cre^+^ mice (*n* = 5 biological replicates per group, scale bar = 200 or 50 µm in EVG staining at different magnifications, scale bar = 2 mm in ultrasound images). (E) Representative terminal deoxynucleotidyl transferase dUTP nick end labelling (TUNEL)‐stained images of *Atf3* ^fl/fl^; *Tmem173* ^fl/+^; *Tagln*‐Cre^+^ mice and *Atf3* ^fl/fl^ *Tagln*‐Cre^+^ mice. Quantification data showing that the number of apoptotic aortic VSMCs was reduced in *Atf3* ^fl/fl^; *Tmem173* ^fl/+^; *Tagln*‐Cre^+^ mice compared with *Atf3* ^fl/fl^ *Tagln*‐Cre^+^ mice (*n* = 4 biological replicates per group, scale bar = 20 or 5 µm at different magnifications) (M: media; A: adventitia). (F) Representative western blot data showing that the cGAS–STING pathway was down‐regulated, cell apoptosis were relieved and contractile phenotype was restored in ATF3 deficiency VSMCs after administration of C‐176 (*n* = 3 biological replicates per group). (G) Flow cytometry analysis showing that administration of C‐176 alleviated cell apoptosis caused by silencing ATF3 with small interfering RNA (*n* = 4 biological replicates per group). The Fisher exact test was used for B, Mann–Whitney test (two‐sided) was performed for D. Unpaired two‐tailed *t*‐test was used in E, F and G. US = ultrasound, *p < .05, **p < .01, ***p < .001. ****p < .0001. MFI, mean fluorescence intensity.

To further determine the effect of ATF3 deletion on VSMCs mediated by the cGAS–STING pathway, we generated VSMC‐specific STING conditional knockout mice based on VSMC ATF3 cKO mice. We found that knocking out one of the STING alleles had a significant rescue effect. In ATF3 fl/fl; Tmem173 fl/+; Tagln‐Cre^+^ mice, there was a significant reduction in AAD incidence (20%, *p* = .0253). Moreover, all mice survived until the end of the experiments (Figure 9A–C). Histological analysis showed that ATF3 fl/fl; Tmem173 fl/+; Tagln‐Cre^+^ mice maintained normal aortic architecture, an intact SMC layer, and exhibited less elastic fibre fragmentation compared with Atf3 cKO mice (Figure 9D). Additionally, the number of TUNEL‐positive VSMCs and the levels of p‐STING and p‐IRF3 in the aortic lesions were markedly down‐regulated in ATF3 fl/fl; Tmem173 fl/+; Tagln‐Cre^+^ mice (Figures 9E and S16C,D). Importantly, VSMC contractile proteins (SM22 and Calponin) were restored in ATF3 fl/fl; Tmem173 fl/+; Tagln‐Cre^+^ mice (Figure S16G,H). Western blot analysis from in vitro experiments indicated that C‐176 administration reduced cGAS–STING pathway activation and caspase‐3 cleavage, while restoring contractile proteins in VSMCs with ATF3 knockdown (Figure 9F). Additionally, the aggravated VSMC apoptosis caused by ATF3 silencing was alleviated with the administration of C‐176 (Figure 9G).

This study indicates that inhibiting the cGAS–STING pathway can partially alleviate the negative impact of ATF3 knockdown on VSMCs.

## 4 DISCUSSION

AAD is a life‐threatening cardiovascular condition that requires surgical intervention. Therefore, identifying therapeutic targets to prevent disease progression is crucial. This study demonstrates that VSMCs consistently exhibit elevated ATF3 expression, which is linked to their inflammatory and apoptotic outcomes.

ATF3 dysfunction is associated with several pathophysiological responses, including inflammation, apoptosis, oxidative stress and endoplasmic reticulum stress, as well as diseases such as cardiovascular diseases.^19^ Additionally, ATF3 is known to promote ferroptosis.^20^ As a downstream target of Ncor1, ATF3 can prevent vascular matrix degradation by inhibiting MMP‐12 and MMP‐13 expression.^21^ However, its effect and mechanism in VSMCs in the context of AAD remain unexplored. To investigate the impact of ATF3 on VSMCs, we knocked out ATF3 in mouse VSMCs. Our results showed that Ang II‐infused (2000 ng/kg/min) Atf3 cKO mice developed more severe AAD and aortic rupture compared with the littermate control group. Additionally, aortic tissue from Atf3 cKO mice exhibited increased VSMC apoptosis. In cultured primary VSMCs, ATF3 knockdown promoted cell damage, increased the release of double‐stranded DNA into the cytoplasm, activated the cGAS–STING pathway and induced both phenotypic switching and apoptosis. These findings suggest that ATF3 is a novel and key molecule in maintaining VSMC function.

The protective effect of ATF3 on smooth muscle is multifaceted. On one hand, ATF3 inhibits the activation of the cGAS–STING pathway, thereby reducing smooth muscle apoptosis caused by DNA damage. On the other hand, ATF3 helps maintain the contractile phenotype of smooth muscle and prevents the phenotypic switch of VSMCs. Research indicates that STING and IRF3 facilitate apoptosis and necroptosis via the type I IFN and tumor necrosis factor receptor pathways. Inhibiting the cytosolic DNA‐sensing STING signalling pathway can prevent phenotypic changes in VSMCs, maintain their function and hinder the development and progression of aortic aneurysms and dissections.^22^ ATF3 acts as an inhibitor of the cGAS–STING pathway by enhancing genomic stability and reducing micronuclei production, thereby mitigating STING and IRF3 phosphorylation. Meanwhile, ATF3 directly inhibits the transcription of STING in VSMCs (Figure 5D,E). ATF3 has also been shown to transcriptionally regulate Tmem173 in macrophages.^23^ Studies indicate that IRF3, a pro‐inflammatory TF activated by cytoplasmic DNA, plays a crucial role in transitioning aortic VSMCs from a contractile to an inflammatory phenotype.^24^ ATF3 inhibits the cGAS–STING pathway, reducing IRF3 activation, which may help preserve the smooth muscle contractile phenotype.

The cGAS–STING pathway is also an important regulator of senescence.^29^, ^30^ In unchallenged, aged Atf3 cKO mice, we observed signs of senescence (Figure S15A,E). In vitro experiments also showed that ATF3 deficiency promotes VSMC senescence (Figure S15F,G). Therefore, we speculate that the absence of ATF3 facilitates both VSMC apoptosis and senescence.

The down‐regulation of the DNA damage repair response promotes the release of nuclear DNA into the cytoplasm in various forms, such as micronuclei and chromosome fragmentation. Increased micronuclei formation due to DNA damage leads to DNA exposure in the cytoplasm, activating the cGAS–STING pathway.^25^, ^26^, ^27^, ^28^, ^29^, ^30^ Thus, effective induction of DNA damage repair reduces the negative effects in VSMCs. ATF3 is essential for genomic stability during genotoxic stress,^31^ and stabilises P21, aiding in DNA damage repair. In VSMCs, ATF3 knockdown leads to cell cycle activation and up‐regulation of genes involved in the G1/S transition, suggesting G1/S cell checkpoint dysfunction and a greater likelihood of VSMCs progressing into the S phase. We explored the relationship between ATF3 and P21 in both in vivo and in vitro analyses. P21, a cyclin‐dependent kinase inhibitor, is crucial for the G1/S cell checkpoint, arresting the cell cycle to enable DNA repair after damage.^32^, ^33^, ^34^, ^35^ Studies have shown that Cdkn1a‐null mouse embryonic fibroblasts exhibit increased DNA damage when exposed to DNA‐damaging agents,^36^ and P21 deficiency leads to enhanced apoptosis.^37^ The regulation of P21 levels significantly affects the fate of VSMCs following chemotherapy and in response to DNA damage.^38^

In our in vitro experiments, we found that silencing ATF3 led to a significant reduction in P21. To further investigate whether ATF3's effect on P21 is mediated by P53, we overexpressed ATF3 in P53‐null H1299 cells.^39^, ^40^ The changes in P21 levels were consistent with alterations in ATF3, indicating that ATF3 can directly regulate P21 independently of P53. Additionally, knocking down ATF3 did not have a prominent effect on P21 mRNA levels. Research indicates that P21 is primarily degraded via the ubiquitin‐proteasome pathway. We found that increasing ATF3 significantly prolonged the half‐life of P21, while the loss of ATF3 accelerated its degradation. However, our co‐IP experiment showed that ATF3 could not directly bind to P21. Thus, we speculated that other molecules might play an intermediary role in this process. Using IP‐mass spectrometry, we identified MDM2 as a potential mediator of ATF3's regulatory effect on P21. Our experiment confirmed that overexpressing ATF3 significantly reduced the binding of MDM2 to P21. Elevated ATF3 levels significantly decreased P21 ubiquitination. Therefore, ATF3 may act as a ‘protector’ of P21 to some extent. When cellular DNA is damaged, ATF3 is rapidly induced to compete with P21 for binding to MDM2, thereby helping P21 evade degradation by MDM2 and indirectly preserving P21 levels.

The function of P21 is highly sophisticated as it needs to respond promptly to DNA damage. If the level of P21 is too high, it can completely block the cell cycle and result in cell senescence. Insufficient P21 permits damaged cells to bypass the G1/S checkpoint, resulting in DNA damage accumulation and increased micronuclei production, which subsequently activates the cGAS–STING pathway. Our unpublished data indicate that peak ATF3 expression decreases with age in mice subjected to identical levels of oxidative stress. This finding suggests that decreased cellular responsiveness to oxidative stress may render middle‐aged and elderly individuals more susceptible to AAD. Further studies are needed to investigate this possibility.

This study highlights the role of ATF3 in VSMCs in protecting against the progression of AAD. The study also found that ATF3 deficiency leads to P21 degradation via ubiquitination, resulting in impaired DNA repair responses and the leakage of damaged DNA into the cytoplasm. This consequently activates the cGAS–STING pathway, leading to the phenotypic switch and apoptosis of VSMCs. Importantly, either pharmacological complementation of P21 function or STING knockdown alleviates ATF3 deficiency‐induced AAD. Therefore, enhancing ATF3 expression in VSMCs could potentially prevent the development of AAD.

In line with previous studies, our research demonstrated that ATF3 deficiency promotes VSMC phenotypic switching and apoptosis, thereby accelerating AAA progression. However, there are several key differences between our study and earlier ones. First, previous studies demonstrated a dynamic pattern in which ATF3 levels initially increase and then decrease following Ang II challenge, providing a more temporal perspective on ATF3's role in the aorta at different time points. In contrast, our study revealed that ATF3 knockout aggravates various multi‐segmental aortic pathologies (TAAA, TAAD, AAAA and AAAD), offering spatial evidence for ATF3's regulatory role in the aorta. Mechanistically, earlier research described ATF3's role as a TF in regulating VSMCs, while our study uncovered ATF3's functions independent of its transcriptional activity. Thus, from multiple perspectives, our study complements previous findings, providing a more comprehensive understanding of ATF3's regulatory role in AAD.

Despite its crucial role in maintaining the homeostasis of smooth muscle cells, ATF3, as a TF, engages in numerous additional downstream pathways. Moreover, ATF3 can exert functions beyond its transcriptional activity by modulating the activity of various proteins. Consequently, designing drugs that target ATF3 could potentially disrupt other pathways, leading to side effects and thus compromising its clinical translational efficacy. Furthermore, ATF3 is differentially expressed across a wide variety of cell types, where it performs diverse biological functions. Therefore, when designing therapeutic drugs for AAD that target ATF3, it is essential to consider the cellular specificity of the treatment. Additionally, single‐cell sequencing data show that ATF3 is highly expressed in arterial fibroblasts following Ang II infusion, but the specific role of ATF3 in fibroblasts requires further experimental validation.

On 30 April, researchers from another institution published an excellent study demonstrating the effect of ATF3 on smooth muscle fate at different stages of AAA (PMID: 38686580).^41^ We were disappointed to have been scooped. Moreover, an earlier study also reported that ATF3 regulates VSMC survival and migration in vitro (PMID: 21280179). Nevertheless, there are significant differences between our findings and previous studies:

1. Their study found that ATF3 plays a regulatory role in AAA, while our study revealed that ATF3 regulates both the thoracic and abdominal aorta, particularly in the ascending aorta and renal ostia. Moreover, the absence of ATF3 not only exacerbates the formation of aortic aneurysms but also leads to aortic dissection in Ang II‐challenged mouse models.
2. Their study explored the role of ATF3 in different stages of AAA using an Ang II‐challenged model. In contrast, our study employed both the Ang II‐challenged model and the PPE‐induced AAA model to examine ATF3's role in VSMCs. We demonstrated that the natural loss of ATF3 results in accelerated aortic aging and the spontaneous development of AAD in aged ATF3cKO mice.
3. Mechanistically, their study illustrated the regulatory role of ATF3 as a TF in VSMCs, whereas we revealed its role independent of transcriptional activity—specifically, in maintaining genomic DNA stability.
4. Our study shows that ATF3 can directly regulate P21 without relying on its interaction with p53. Mechanistically, ATF3 may enhance the stability of P21 by down‐regulating MDM2‐mediated ubiquitination of P21.
5. Our research demonstrated the regulatory role of ATF3 in the cGAS–STING pathway within VSMCs and revealed that ATF3 can regulate the phenotype of VSMCs via the cGAS–STING pathway.
6. Additionally, our study demonstrated the therapeutic potential of roscovitine and C‐176 for AAD, highlighting their clinical translational value and offering new approaches for drug development.
7. Their study used adeno‐associated virus (AAV) as vectors to silence ATF3 expression, while we used mice with ATF3 conditional knockout in VSMCs (Atf3 fl/fl Tagln Cre^+^) to investigate the effects of ATF3 loss on the aorta. AAV does not persist long within the body, which may explain why the researchers from the other institution did not observe spontaneous AAD formation. In contrast, we used ATF3cKO mice to provide a more detailed elucidation of the pathological changes in all segments of the aorta.

Therefore, we humbly believe that our research findings complement previous studies and further clarify the regulatory role of ATF3 in VSMCs, particularly in maintaining DNA stability.

## AUTHOR CONTRIBUTIONS

*Writing—review and editing, writing—original draft, visualisation, validation, methodology, investigation, formal analysis, data curation and conceptualisation*: Y. D. *Writing—review and editing, methodology, investigation and conceptualisation*: P. H. *Writing—review and editing, investigation and formal analysis*: X. D. *Writing—review and editing, investigation and data curation*: D. W. *Writing—review and editing and methodology*: J. L. *Writing—review and editing and investigation*: S. L. *Writing—review and editing and data curation*: L. R. *Writing—review and editing and data curation*: M. C. *Writing—review and editing, supervision, project administration, funding acquisition and conceptualisation*: P. Y. *Writing—review and editing, supervision, project administration, funding acquisition and conceptualisation*: J. X.

## CONFLICT OF INTEREST STATEMENT

The authors declare no conflicts of interest.

## ETHICS STATEMENT

The Union Hospital Independent Ethics Committee at Huazhong University of Science and Technology approved this human tissue study protocol (Ethical approval number UHCT‐IEC‐SOP‐016‐03‐01). The Institutional Animal Care and Use Committee of Huazhong University of Science and Technology approved the animal experiments in this study (IACUC Number 3552). Our study adhered to the US National Institutes of Health's Guide for the Care and Use of Laboratory Animals (8th edition, 2011).

## Supporting information



Supporting Information

Supporting Information

## Data Availability

All data that support the findings of this study are available from the corresponding author upon reasonable request.
