# Supplementary material for: Deficiency of ATF3 facilitates both angiotensin II‐induced and spontaneously formed aortic aneurysm and dissection development by activating cGAS–STING pathway
Source: Clin Transl Med. 2024 Dec 27;15(1):e70147. doi: 10.1002/ctm2.70147 (PMC11680558; doi:10.1002/ctm2.70147)
Supplement: Supplementary file 1 — Supporting Information [file CTM2-15-e70147-s002.docx]

**SUPPLEMENTAL MATERIAL**

**Human Tissue Study**

The patient samples and healthy control sample were provided by the Department of Cardiovascular Surgery, Union Hospital, Tongji Medical College, Huazhong University of Science and Technology, China, in accordance with the regulations of the Ethics Committee of Huazhong University of Science and Technology (Ethical approval number: UHCT-IEC-SOP-016-03-01). All procedures relating to human clinical samples were performed in accordance with the principles of the 1975 Declaration of Helsinki. Patient aortic tissues were collected during aortic repair surgery, and a portion of this discarded tissue is collected. Then we selected the part of the discarded aorta tissue with a relatively intact media layer for our experiment. Control aortic tissue was collected from age-matched organ donors without aortic aneurysm, dissection, coarctation, or previous aortic repair.

The aortic tissue was divided into several segments, which were then fixed in 4% paraformaldehyde, and embedded in paraffin for histologic analysis, placed in RNAlater for RNA analysis, or snap-frozen in liquid nitrogen for protein extraction.

**Animal Studies**

Mice were maintained in the specific pathogen-free facility of the Tongji Medical College, Huazhong University of Science and Technology. All animal experiments and protocols were approved by the Institutional Animal Care and Use Committee of Huazhong University of Science and Technology (IACUC Number: 3552). To generate a mouse model of sporadic AAD, *Atf3* VSMC-specific conditional knockout mice (*Atf3* cKO, C57BL/6J-*Atf3* fl/fl, *Tagln* Cre^+^, male) and the littermate control mice (C57BL/6J-*Atf3* fl/fl, *Tagln* Cre^-^, male) were constructed by Saiye Inc. (Suzhou, China). *Atf3* VSMC-specific conditional knockout with a combination of *Tmem173* knockdown mice (C57BL/6J-*Atf3* fl/fl, *Tmem173* fl/+, *Tagln* Cre^+^, male) were constructed by Model Organisms (Shanghai, China). Sporadic AAD was induced by challenging mice with Ang II infusion. C57BL/6J-*Atf3* fl/fl, *Tagln* Cre^+^ mice were challenged with 2,000 ng/min/kg Angiotensin II (HY-13948, MedChemExpress Corp.) infusion for 4 weeks through an osmotic minipump (Model 2004; ALZET Technical Support, Cupertino, CA). C57BL/6J-*Atf3* fl/fl, *Tagln* Cre^-^ mice infused with 2,000 ng/min/kg Ang II for 4 weeks were used as controls. the mice used in our study were male mice, except where otherwise indicated.

In Ang II infusion assay, Mice were infused with angiotensin II (Ang II) or saline with subcutaneously implanted Alzet osmotic minipumps implanted under isoflurane anaesthesia (2%) by inhalation. Blood pressure was measured by tail-cuff plethysmography at Days 0, 7, 14, 21, and 28 during the Ang II infusion. Measurements were performed at the same time of the day, and at least five individual observations were taken for each animal and averaged.

In rescue experiments for P21, C57BL/6J-*Atf3* fl/fl, *Tagln* Cre^+^ mice were challenged with Ang II as described above and were given either Roscovitine (SelleckChem S1153) 50 mg/kg dissolved in corn oil or corn oil (control) daily by intraperitoneal injection during the Ang II infusion period.

In rescue experiments for STING, C57BL/6J-*Atf3* fl/fl, *Tagln* Cre^+^ mice and C57BL/6J-*Atf3* fl/fl, *Tmem173* fl/+, *Tagln* Cre^+^ mice were challenged with Ang II as described above.

At the end of the challenge period, mice aortic diameters were measured using a doppler ultrasound Vevo 1100 Imaging System (VisualSonics) with a real-time microvisualization scan head in B mode. Then mice were euthanized, and their aortas were exposed, cleaned, extracted, and imaged for disease evaluation. The aortic segments were embedded in 4% paraformaldehyde for histology and immunofluorescence staining or were snap-frozen for protein analysis.

In senescence assay, C57BL/6J-*Atf3* fl/fl, *Tagln* Cre^+^ mice and C57BL/6J-*Atf3* fl/fl, *Tagln* Cre^-^ mice were maintained for 18 months. Then mice were euthanized, and their aortas were exposed, cleaned, extracted, and imaged for disease evaluation. The aortic segments were embedded in 4% paraformaldehyde for histology and immunofluorescence staining or were snap-frozen for protein analysis.

In porcine pancreatic elastase (PPE)-induced abdominal aortic aneurysm (AAA) model, to induce murine AAA, the PPE infusion model was performed as previously described^1^. Mice were supine on the control panel after being anesthetized with a 1% pentobarbital sodium intraperitoneal injection. The vena cava and separated tissues were pushed to each side after careful dissection, allowing circumferential exposure of the infrarenal abdominal aorta. 20-30 μl of elastase (Cat# SLBV9311, E1250, MilliporeSigma) or heat-inactivated elastase was administered externally to the exposed aortic adventitia using a pipette with a fine tip. The exposed region was gently cleansed with regular saline solution and dried with cotton-tip applicators after 40 minutes. The elastase-soaked cotton was then discarded, and the abdominal cavity was cleaned twice with saline before the surgical wound was closed. Fourteen days after elastase exposure or fourteen days after heat-inactivated elastase exposure, the abdominal aorta was extracted. To reduce technical factors, all surgical techniques were carried out by a single surgeon. The abdomen was normally closed, and the mouse was recovered.

Finally, animals were euthanized by CO2 or isoflurane (2%) narcosis. Ethics approval for the animal experiments in this study was obtained from the Institutional Animal Care and Use Committee of Huazhong University of Science and Technology. Our study conformed to the Guide for the Care and Use of Laboratory Animals published by the US National Institutes of Health (NIH Publication, 8th edition, 2011).

**Definition of Aortic Dilatation, Aortic Aneurysm, and Aortic Dissection**

For each aortic segment of mice, dilatation was defined as an aortic diameter ≥1.25 but <1.5 times the mean aortic diameter of the segment in unchallenged mice with the same genetic background. Aneurysm was defined as an aortic diameter ≥1.5 times the mean aortic diameter of the segment in unchallenged mice with the same genetic background. Aortic dissection was defined as the presence of hematoma within the aortic wall detected on gross examination or as the presence of layer separation within the aortic media or medial-adventitial boundary (with a false lumen hematoma) detected upon histologic examination of the aorta.

**Elastic Fiber Staining**

Abdominal aortic tissues were fixed in 4% paraformaldehyde (PFA) and embedded in paraffin. Tissue sections (5 µm thick) were stained with Verhoeff–Van Gieson (Sigma-Aldrich) according to the manufacturer’s instructions. The aortic sections were examined by 2 independent observers who were blinded to the experimental groups. The extent of elastic fiber fragmentation was scored on a scale of 0 to 3 (0 = none, 1 = minimal, 2 = moderate, and 3 = severe).

Tissue sections (5 µm thick) were dewaxed, antigen retrieved, blocked with donkey serum and incubated overnight with primary antibodies. Slides were washed with PBS-Tween20 and incubated with appropriate secondary antibodies.

**Immunofluorescence Staining and Imaging**

Tissue sections (5 µm thick) were dewaxed, antigen retrieved, blocked with donkey serum. The sections or cells were then incubated with primary antibody at room temperature for 2 hours or at 4°C overnight, and were washed with PBS-Tween20 and incubated with appropriate secondary antibodies. Nuclei were counterstained with 4',6-diamidino-2-phenylindole (DAPI). Slides of sections or treated cells incubated with secondary antibody alone were used as negative controls. Tissue sections were examined by using Olympus microscope (OLYMPUS IX2-UCB) or a Nikon C2+ confocal microscope (Nikon).

**TUNEL Assay and Immunofluorescence Staining**

To study apoptosis using an in situ cell death detection kit (C1086, Beyotime, China), we performed TUNEL staining according to the manufacturer’s instructions.

**Annexin V/Propidium Iodide Staining**

Cell death was detected by performing flow cytometry analysis of cells stained with annexin V (apoptotic cell marker) and propidium iodide (PI) (necrotic cell marker). Staining was accomplished with the use of annexin V-FITC/PI Apoptosis Detection Kit (HY-K1073, MedChemExpress). Treated cells were harvested, washed with PBS, and stained with annexin V-FITC and PI in the dark at room temperature for 15 minutes. The samples were examined immediately on a flow cytometer (BD FACSAria^TM^ II), and the data were analyzed by using FlowJo software.

**Western blotting**

Proteins extracted from cells or tissue samples were lysed using RIPA Lysis Buffer (Biosharp, China) and separated by 10% SDS–PAGE gels in the presence of protease and phosphatase inhibitors (Beyotime, China). PVDF membranes (Millipore, Bedford, MA) were used for protein transfer. Subsequently, the membranes were blocked using NcmBlot Blocking Buffer (NCM Biotech, China) for 10 minutes at room temperature, and the primary antibodies were then incubated overnight at 4°C. HRP-conjugated Goat Anti-Rabbit IgG (ABclonal, China) was used as the second antibody. GAPDH or β-actin antibodies were used to assess total protein levels. Protein bands were detected using the JS-1070P chemiluminescence gel imaging system (P&Q Science & Technology, China), and quantified using ImageJ.

**Real-time Quantitative RT-polymerase Chain Reaction (PCR)**

Total RNA from the VSMC was extracted with RNA-easy Isolation Reagent (R701, Vazyme, China) according to the manufacturer’s instructions. The mRNA was reverse-transcribed into cDNA by using the PrimeScript^TM^RT Master Mix (RR036A, Takara, Japan). Real-time PCR was performed by using the Real-Time PCR System (Bio-Rad).

The primers(mouse):

*Nr4a2*:

5-GTGTTCAGGCGCAGTATGG-3(forward)

5-TGGCAGTAATTTCAGTGTTGGT-3(reverse)

*Cebpb*:

5-GACAAGCTGAGCGACGAGTA-3(forward)

5-TGCTTGAACAAGTTCCGCAG-3(reverse)

*Tcf15*:

5-GTGTAAGGACCGGAGGACAA-3(forward)

5-GATGGCTAGATGGGTCCTTG-3(reverse)

*Cdkn1a*:

5-TGTACCGCTATGGTTACACTCG-3(forward)

5-GGCAGGGACAGTTGCTTCT-3 (reverse);

*Atf3:*

5-GTAACC CGTTGAACCCCATT-3 (forward)

5-CCATCCAATCGGTAGTAGCG-3 (reverse)

*Tmem173:*

5-GGCTGGCCTGGTCATACTAC-3 (forward)

5-GCACCACTGAGCATGTTGTT-3(reverse)

*Il6:*

5-TAGTCCTTCCTACCCCAATTTCC-3 (forward)

5-TTGGTCCTTAGCCACTCCTTC-3 (reverse)

*Il1β:*

5-GCAACTGTTCCTGAACTCAACT-3 (forward)

5-ATCTTTTGGGGTCCGTCAACT-3 (reverse)

*Tnf-α:*

5-ACCCTCACACTCAGATCATCTTC-3 (forward)

5-TGGTGGTTTGCTACGACGT-3 (reverse)

*Gapdh:*

5-GTCATCCCAGAGCTGAACG-3 (forward)

5-TCATACTTGGCAGGTTTCTCC-3 (reverse)

The mRNA levels were acquired from the value of the threshold cycle (Ct) and were normalized against the Ct of GAPDH.

**Co-immunoprecipitation (Co-IP)**

The protein A/G-agarose beads (Santa Cruz, CA) were incubated with normal IgG, P21, ATF3, MDM2, Flag or His antibody at 4°C for 2 hours. HEK-293T cells were lysed in 500 µl Cell lysis buffer for Western and IP(P0013, Beyotime Biotechnology) on ice for 5 min, and the supernatants were incubated with antibody-conjugated beads at 4°C overnight. After washing with the Co-IP buffer, proteins were eluted from the beads and boiled in SDS loading buffer. Western blotting was performed to detect the precipitation of proteins.

**Ubiquitination Assay**

The protein A/G-agarose beads (Santa Cruz, CA) were incubated with P21 antibody at 4°C for 2 hours. 293T cells or VSMCs were lysed in 500 µl Co-IP lysis buffer (P0013, Beyotime Biotechnology) on ice for 5 min, and the supernatants were incubated with antibody-conjugated beads at 4°C overnight. After washing with the Co-IP buffer, proteins were eluted from the beads and boiled in SDS loading buffer. Western blotting was performed to detect the ubiquitination of proteins.

**RNA sequencing and data analysis**

Total RNA was extracted from cell or tissue samples using a RNeasy kit (QIAGEN, 74004). After quality control, cDNA synthesis, end-repair, A-base addition, and ligation of the Illumina indexed adapters were performed according to the instructions for the TruSeq PE Cluster Kit v3-cBot-HS (Illumina, PE-401-3001). The concentration and size distribution of the completed libraries were assessed on the Agilent Bioanalyzer 2100 system. RNA-seq libraries were sequenced using a NovaSeq 6000 with paired-end 150-cycle reads. The sequenced samples were analyzed using the Subjunc-FeatureCounts-DESeq2 pipeline on our bioinformation server with Ubuntu 20.04 LTS. Clean reads were mapped to the mm10 (UCSC) mouse genome with Subjunc (v2.0.1) with default parameters. Transcripts were assembled, and expression was quantified using featureCounts (v2.0.1) with mouse annotation (release_M20) from Gencode. Differentially expressed genes were analyzed using the DESeq2 package (v1.30.1) of R v4.0.4 software. Genes with less than five percent probability to be false positive (p-adjusted < 0.05) and with absolute log2 (fold change) > 1 or 0.5 were chosen for downstream analysis and subsequent functional analyses. The volcano plot was plotted in R with ggplot2. The heatmap was plotted in GENE-E (Broad Institute). The MA plot was generated with the ‘plotMA’ function of DESeq2. Gene set enrichment analysis (GSEA) was performed with the ‘GSEA’ function in clusterProfiler v3.18.1. The hallmark gene sets from the Molecular Signatures Database (MSigDB) were obtained via msigdbr (v7.5.1). The P values of the enriched pathways were then adjusted using the BH method, with the threshold set to p-adjusted <0.05.

**Single-cell RNA-Seq and Bioinformatics Analysis**

The single cells were processed via the GemCode Single Cell platform using GemCode Gel Bead Kit and Chips and Library Kits (Cat #1000121, America, 10x Genomics) according to the manufacturer’s protocol. In brief, single cells were sorted into 0.4% BSA-PBS solution, about 10000 cells were added to each channel, and 5000 or 10000 cells were captured in different samples. Single-Cell RNA Sequencing workflow, such as single-cell capture, library preparation, quality control PCR, and sequencing, were performed at Stanford Genome Sequencing Service Center. The cells were then partitioned into gel beads to generate barcoded Gel Bead in emulsion by using a 10x Genomics microfluidics chip in the GemCode instrument according to the manufacturer’s protocols, in which cell lysis and barcoded reversed transcription of RNA, then amplification, shearing, and 5′ adaptor and sample index attachment were performed. Libraries were created, and samples were multiplexed and sequenced on an Illumina NextSeq 500 system by following10× Genomics protocols.

The Cell Ranger single-cell software suite 3.1.0 (https://www.10xgenomics.com/) was utilized to convert the sequence data into expression matrices on the High-Performance Computing System (AMD Ryzen 9 3950X 16-Core, 32-Thread, 128GB RBM). The raw base-calling data were demultiplexed on the HiSeq4000 sequencer to generate FASTQ files via the cellranger mkfastq pipeline. Subsequently, the mouse transcriptome (mm10-3.0.0) was sequenced, and the reads were aligned. We employed the cellranger count pipeline to correct the cell barcodes and the unique molecular identifiers (UMI) to obtain the expression matrices. Then we imported final output filtered expression matrices into the Seurat package (version 3.6.3) in R and used the CreateSeuratObject function to create Seurat objects that retained only genes detected in >3 cells and cells with cells >200 distinct genes. The threshold for individual cell read counts (nCount_RNA) was set between 1000 to 45000; genes (nFeature_RNA) were set between 500 to 4500; mitochondrial gene content was set below 10% to exclude doublets/clumps and free RNA. Subsequently, data normalization, scaling, and regression by mitochondrial were performed using default settings in Seurat. Then, we performed a principal component (PC) analysis for dimensional reduction with Seurat functions based on the variable genes previously identified.

Cell population deconvolution was performed by the dimensional reduction and shared nearest neighbor (SNN) modularity clustering algorithm. We defined the cell clusters based on a set of highly expressed differential/conservative/cell marker genes.

differential/conservative/cell marker genes. DEGs in each cluster across different conditions were identified by using the “FindMarkers” function in Seurat. Within each cluster, DEGs between two groups of cells were identified by using a Wilcoxon rank sum test. Adjusted p-values were calculated on the basis of the Bonferroni correction by using all features in the dataset. Genes with a p_val_adj < 0.05 were considered as DEGs.

**Cell Culture and Transfection**

Aortic smooth muscle cells were purchased from Procell (CP-M076, Wuhan, China) and were cultured in Dulbecco’s Modified Eagle’s Medium (DMEM; Gibco, United States) supplemented with 10% fetal bovine serum (FBS, ES Cell Qualified New Zealand Origin; Gibco, United States) and 1% penicillin-streptomycin in a humidified atmosphere containing 5% CO2 at 37°C. VSMCs were transfected with *Atf3* siRNA (target sequence: 5′-GCATCCTTTGTCTCACCAATT-3′) or Control siRNA(target sequence: 5′- CCTAAGGTTAAGTCGCCCTCG-3′) followed by treatment with 500 µM H2O2. *Atf3* siRNA or Control siRNA were constructed by OBiO Technology (Shanghai, China). *Mdm2* siRNA (Forward: 5′-GGAAAAUAUAUGCAAUGAUCUA-3′, Reverse: 5′-UAGAUCAUUGCAUAUAUUUUCC-3′)

In P21 rescue experiments, aortic smooth muscle cells were transfected with *Atf3* siRNA or Control siRNA, then cells were incubated in the absence of Roscovitine or DMSO, followed by treatment with 500 µM H2O2.

In STING rescue experiments, aortic smooth muscle cells were transfected with *Atf3* siRNA or Control siRNA, then cells were incubated in the existence of C-176(Selleckchem S6575) or DMSO, followed by treatment with 500 µM H2O2.

**Isolation and Culture of Mouse Primary Aortic Smooth Muscle Cells**

Primary aortic smooth muscle cells (VSMCs) were isolated from the aortas of *Atf3* cKO mice and littermate control mice, as previously described.^2^ Primary VSMCs were grown to confluence in DMEM (Gibco) supplemented with 10% fetal bovine serum (Biological Industries), 100 U/mL penicillin, and 100 μg/mL streptomycin at 37 °C in 5% CO2.

**Cell senescence model**

To induce senescence of VSMCs, cells were starved at 0.1% FBS DMEM medium overnight to achieve synchronization and then Ang II (0.3μM, HY-13948, MedChemExpress Corp.) was added into and cultured for 5 days. Medium was changed every day to prevent loss of function of Ang II.

**Senescence-associated-β-galactosidase (SA-β-gal) staining**

Cultured cells or aorta samples were washed in ice-cold PBS buffer for three times and then fixed in 1% glutaraldehyde (Sigma) for 15 min at room temperature. After washing for three times, the cells or aortae were incubated in staining buffer (X-gal concentration: mg/ml) for 12-hours (for cells) or 24 hours (for aortae) at 37oC. SA-β-gal images were obtained with light microscope (cultured cells) or camera (tissue samples), then analyzed with Image J software (NIH, Bethesda). The degree of the senescence (blue-green) was evaluated according to the grading score system.

**Statistical Analyses**

Representative figures and images are used to illustrate typical outcomes of each experiment. The Shapiro-Wilk test for normality and the Brown-Forsythe test for equality of group variance were performed on all data using Prism 9 software (GraphPad Software Inc., La Jolla, CA). Differences between two groups were evaluated using an unpaired, two-tailed Student’s t-test for normally distributed data, or a Mann-Whitney test for non-normally distributed data. One-way ANOVA was used to assess differences across groups for a single independent variable in multiple comparisons, while a two-way ANOVA was used to examine differences across groups for two independent variables. P-values for pairwise comparisons were adjusted using the Bonferroni method when indicated. The figure legends specify the statistical tests used. Each experiment was conducted a minimum of three times. All data points represent independent samples, not technical replicates. Data are expressed as mean ± standard deviation (SD).


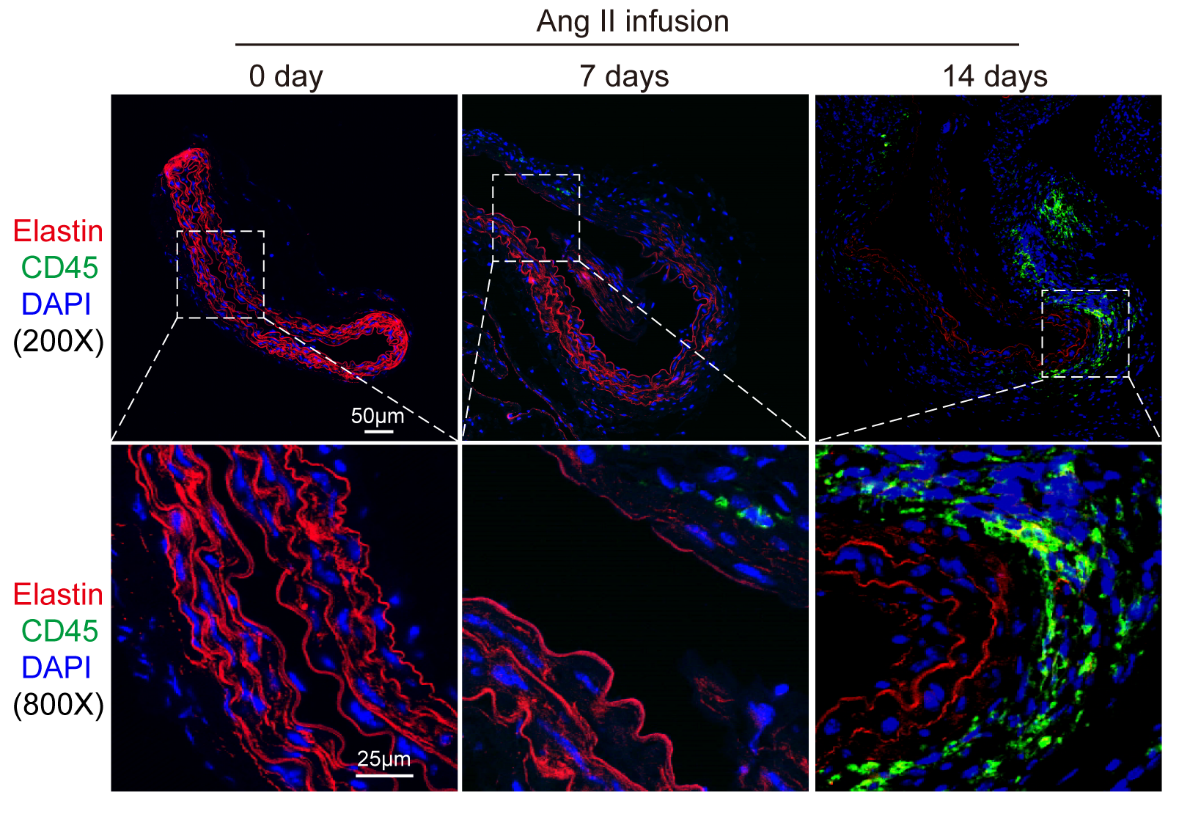
 **Supplemental Figure1**

The infiltration of immune cells in the aorta at 0, 7, and 14 days after Ang II perfusion.


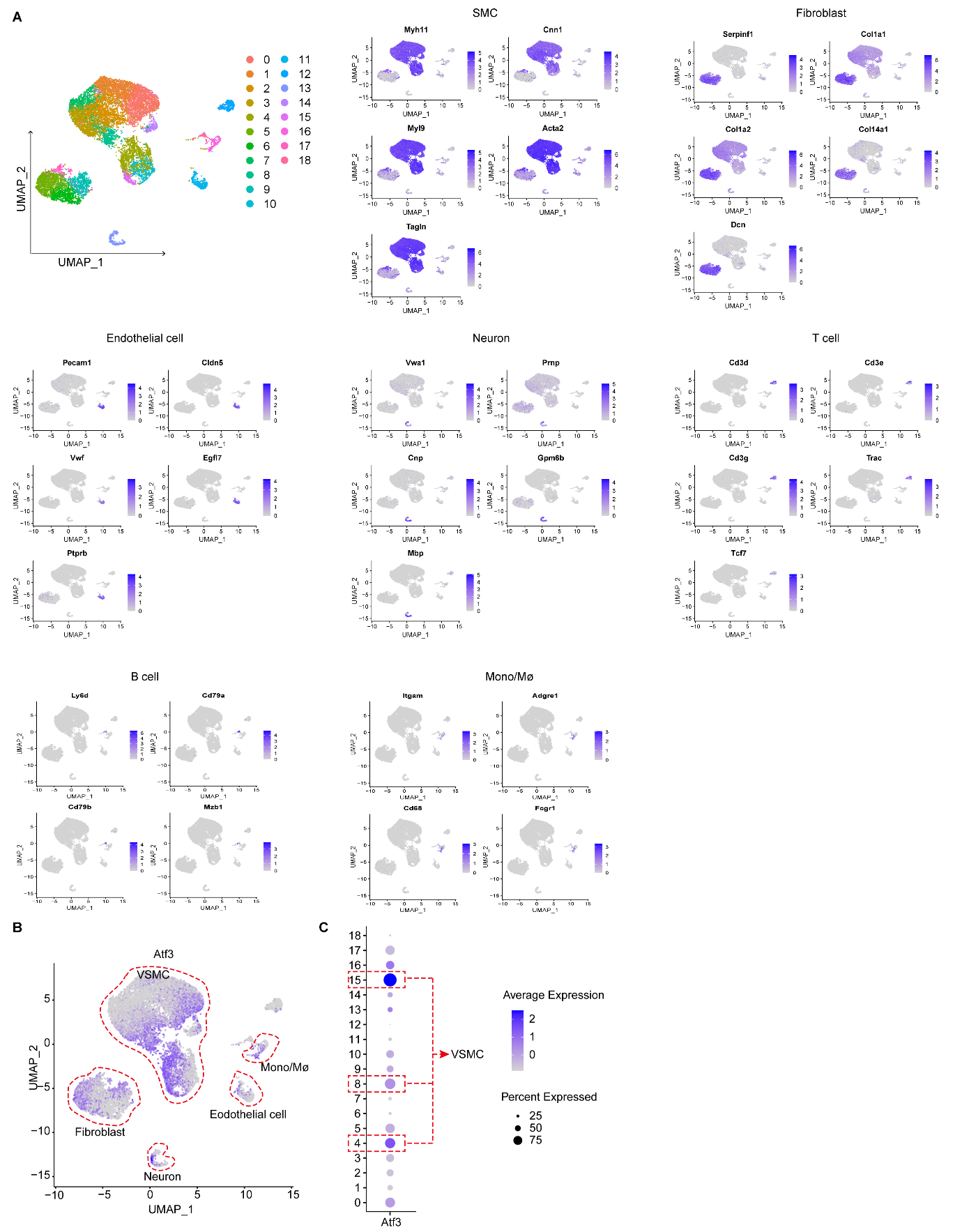


| **Clusters** | **0,1,2,3，4,7,8,10,14,15** | **5,6,9,17** | **11** | **12** | **18** | **16** | **13** |
| --- | --- | --- | --- | --- | --- | --- | --- |
| **Cell type** | **Vascular smooth**  **muscle cell** | **Fibroblast** | **Endothelial cell** | **T**  **cell** | **B cell** | **Monocyte/ Macrophage** | **Neuron** |

**Supplemental Figure2.** **A,** Feature plot of markers from different aortic subclusters.

**B-C,** ATF3 expression in different subclusters.


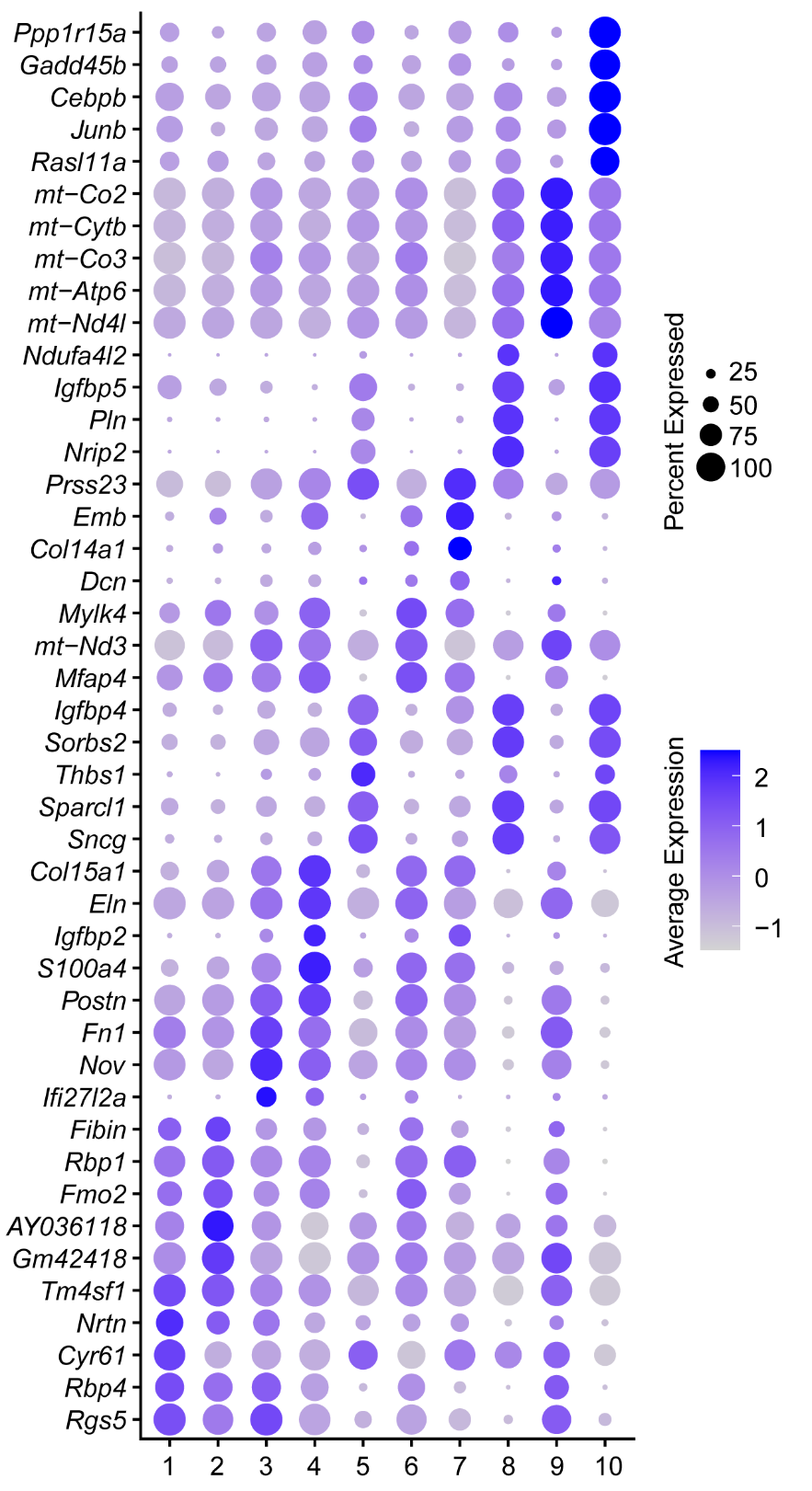


**Supplemental Figure3**

Dot plot representing the cluster-specific differentially expressed genes (DEGs).
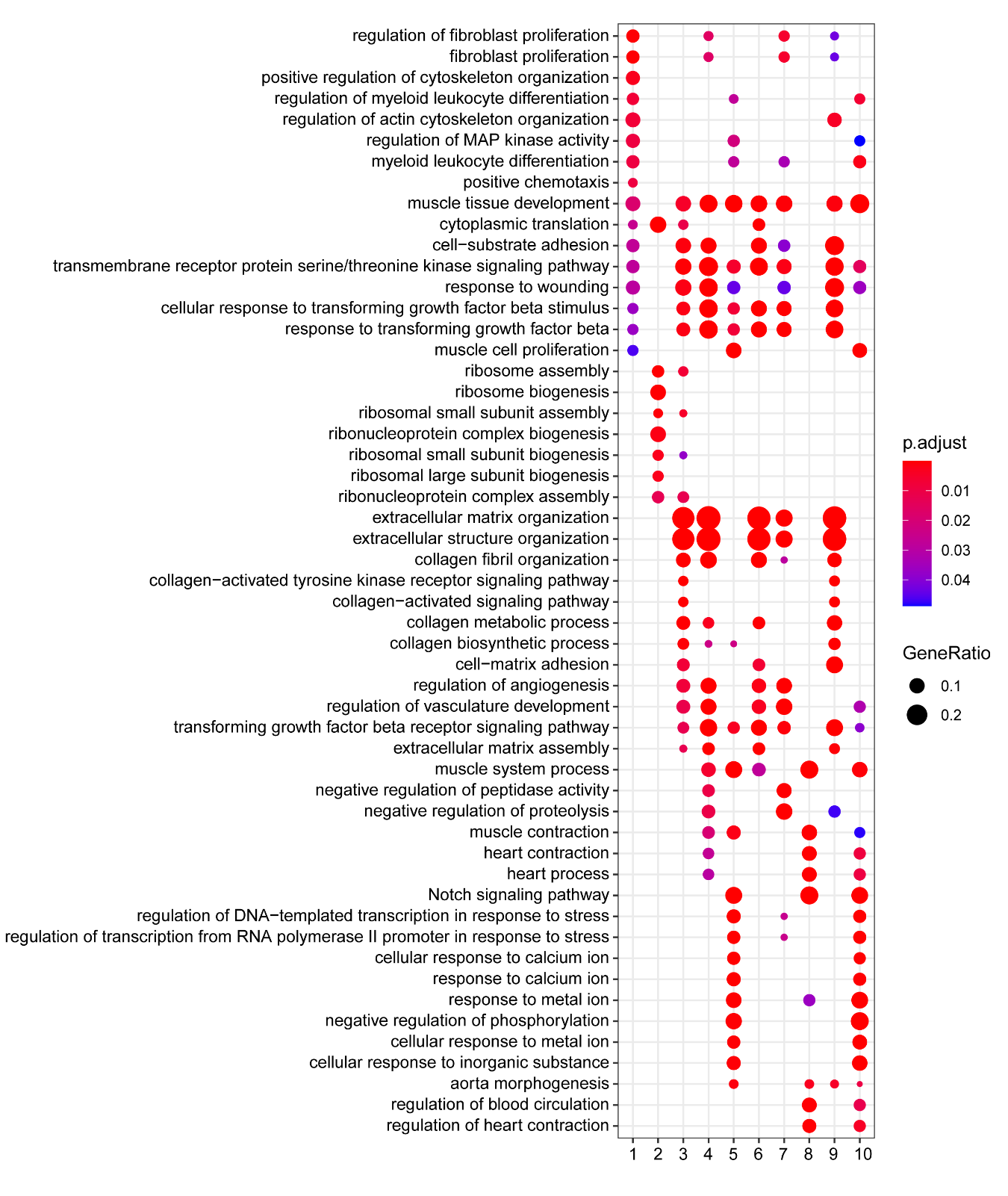


**Supplemental Figure4**

Gene ontology (GO) analysis on cluster-specific DEGs to identify the overrepresented functions.


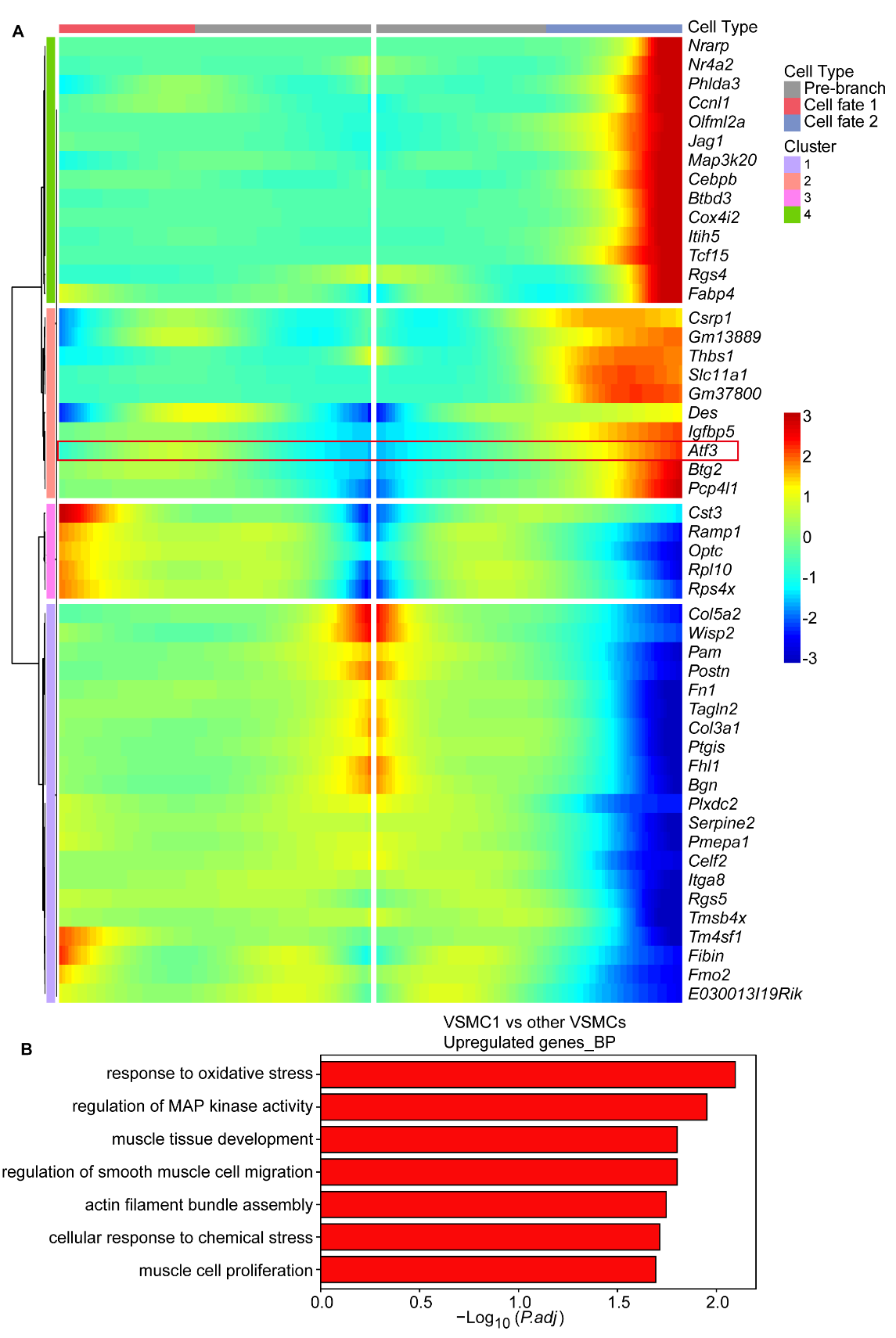


**Supplemental Figure 5. Branch-dependent expression analysis in node1**.

A. Differentially expressed genes driving VSMC differentiation (Top50). ATF3 is specifically highlighted. B. Gene ontology (GO) analysis identified Vsmc1 as stressed/proliferative VSMCs.


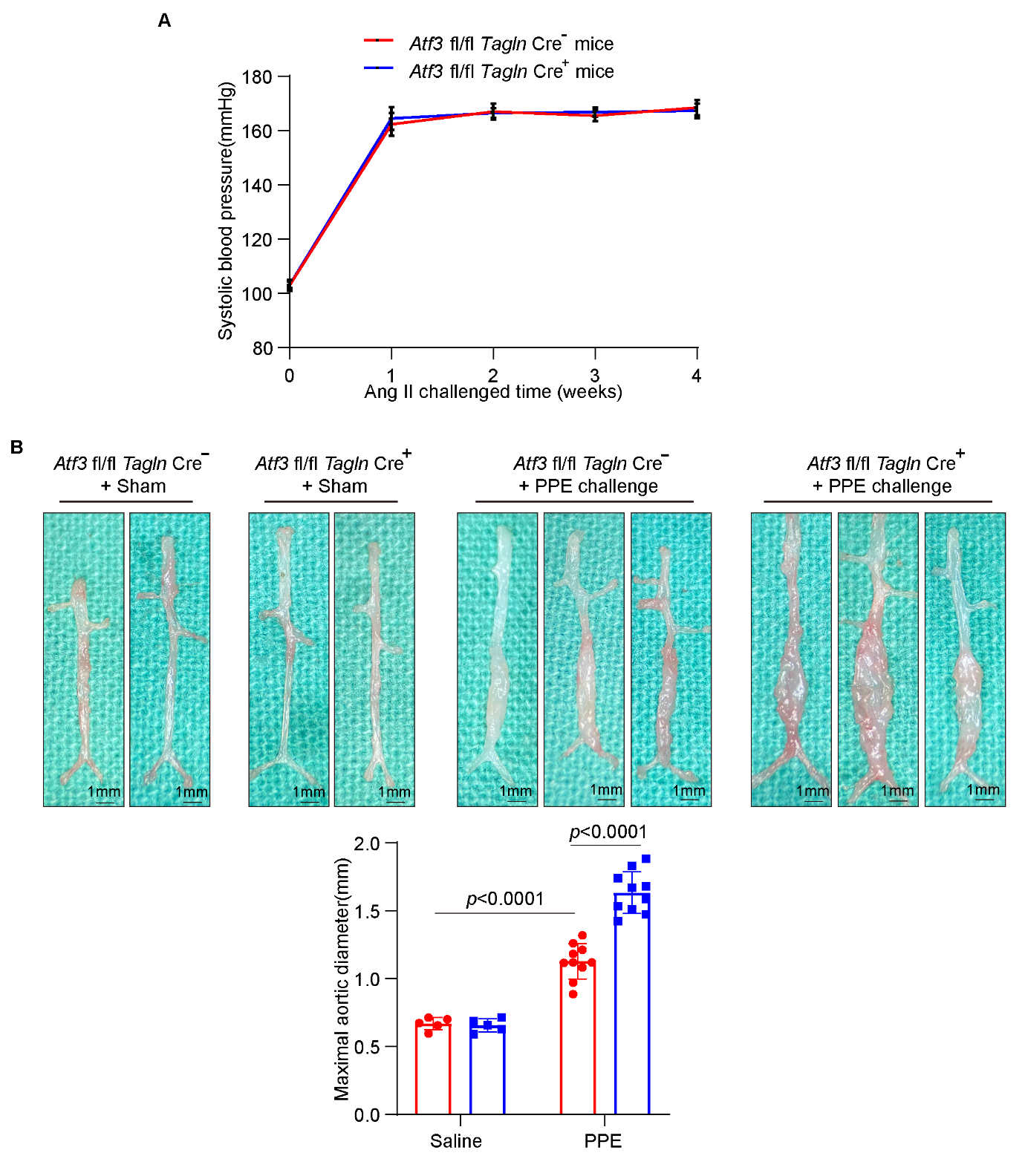


**Supplemental Figure 6. A,** *Atf3* cKO mice and littermate control mice were challenged with Ang II infusion (2000 ng/min/kg) for 4 weeks. Systolic blood pressure was similarly increased in *Atf3* cKO mice and littermate control mice challenged with Ang II (n=6 biological replicates per group). **B**, *Atf3* cKO mice and littermate control mice were unchallenged or challenged with porcine pancreatic elastase (PPE). Representative images of excised aortas showing more severe aortic enlargement in PPE-challenged *Atf3* cKO mice than in littermate control mice (n=5 biological replicates in saline treated groups, n=10 biological replicates in PPE challenged groups, scale bar=1mm). Two-way ANOVA with the Bonferroni post-hoc test for pairwise comparisons was used for **B**.


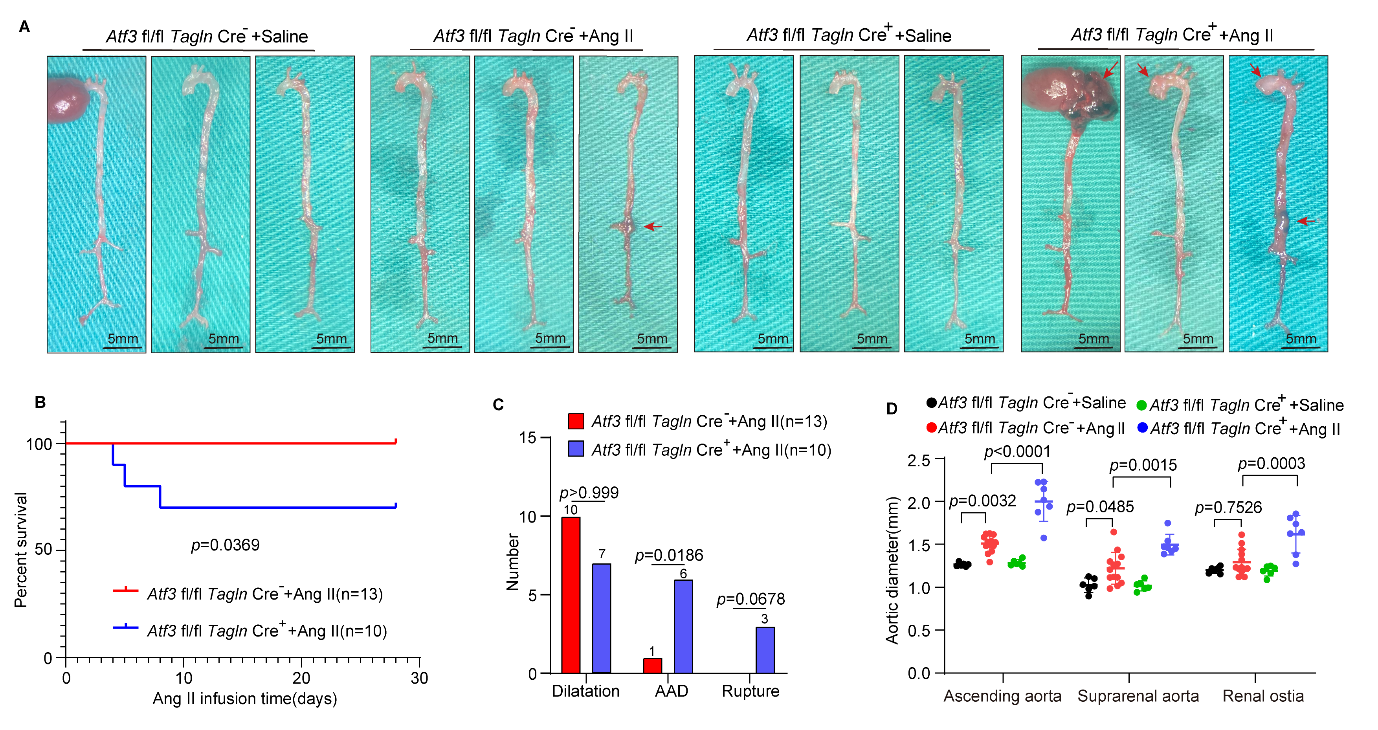


**Supplemental Figure 7. Remodeling of *Atf3* cKO mice.**

**A**, Representative images of excised aortas showing severe aortic enlargement and AAD formation in Ang II challenged *Atf3* cKO mice than in Ang II challenged littermate control mice (n=13 biological replicates in *Atf3* fl/fl *Tagln* Cre^-^+Ang II group, n=10 biological replicates in *Atf3* fl/fl *Tagln* Cre^+^+Ang II group, scale bar=5mm). **B**, Kaplan-Meier survival analysis showing aggravated survival in challenged *Atf3* cKO mice compared with challenged littermate control mice during the 28 days of Ang II infusion. **C**, the incidence of AAD was significantly higher in challenged *Atf3* cKO mice than in littermate control mice.  **D**, Mean aortic diameters of various aortic segments were larger in challenged *Atf3* cKO mice than in littermate control mice. The measurements were based on the excised aortas (n=6 biological replicates in *Atf3* fl/fl *Tagln* Cre^-^+Saline or *Atf3* fl/fl *Tagln* Cre^+^+Saline group, n=13 biological replicates in *Atf3* fl/fl *Tagln* Cre^-^+Ang II group, n=7 biological replicates in *Atf3* fl/fl *Tagln* Cre^+^+Ang II group).


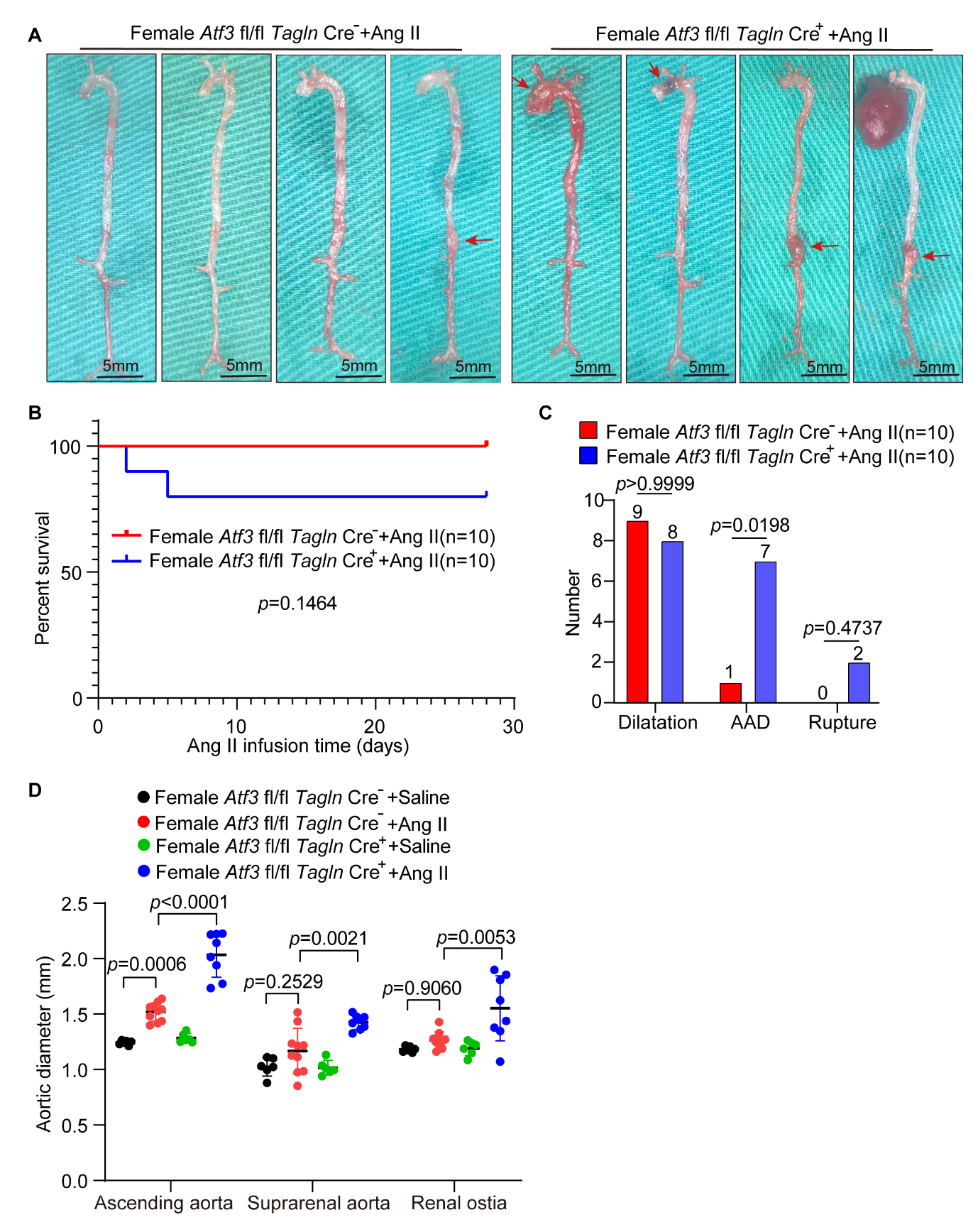


**Supplemental Figure8.Ang II (2000ng/kg/min) challenged female *Atf3* cKO mice.**

**A**, Representative images of excised aortas showing severe aortic enlargement and AAD formation in Ang II challenged female *Atf3* cKO mice than in Ang II challenged female littermate control mice (n=10 biological replicates per group, scale bar=5mm). **B**, Kaplan-Meier survival analysis showing aggravated survival in challenged female *Atf3* cKO mice compared with challenged female littermate control mice during the 28 days of Ang II infusion. **C**, the incidence of AAD was significantly higher in challenged female *Atf3* cKO mice than in female littermate control mice.  **D**, Mean aortic diameters of various aortic segments were larger in challenged female *Atf3* cKO mice than in female littermate control mice. The measurements were based on the excised aortas (n=6 biological replicates in *Atf3* fl/fl *Tagln* Cre^-^+Saline or *Atf3* fl/fl *Tagln* Cre^+^+Saline group, n=10 biological replicates in *Atf3* fl/fl *Tagln* Cre^-^+Ang II group, n=8 biological replicates in *Atf3* fl/fl *Tagln* Cre^+^+Ang II group).


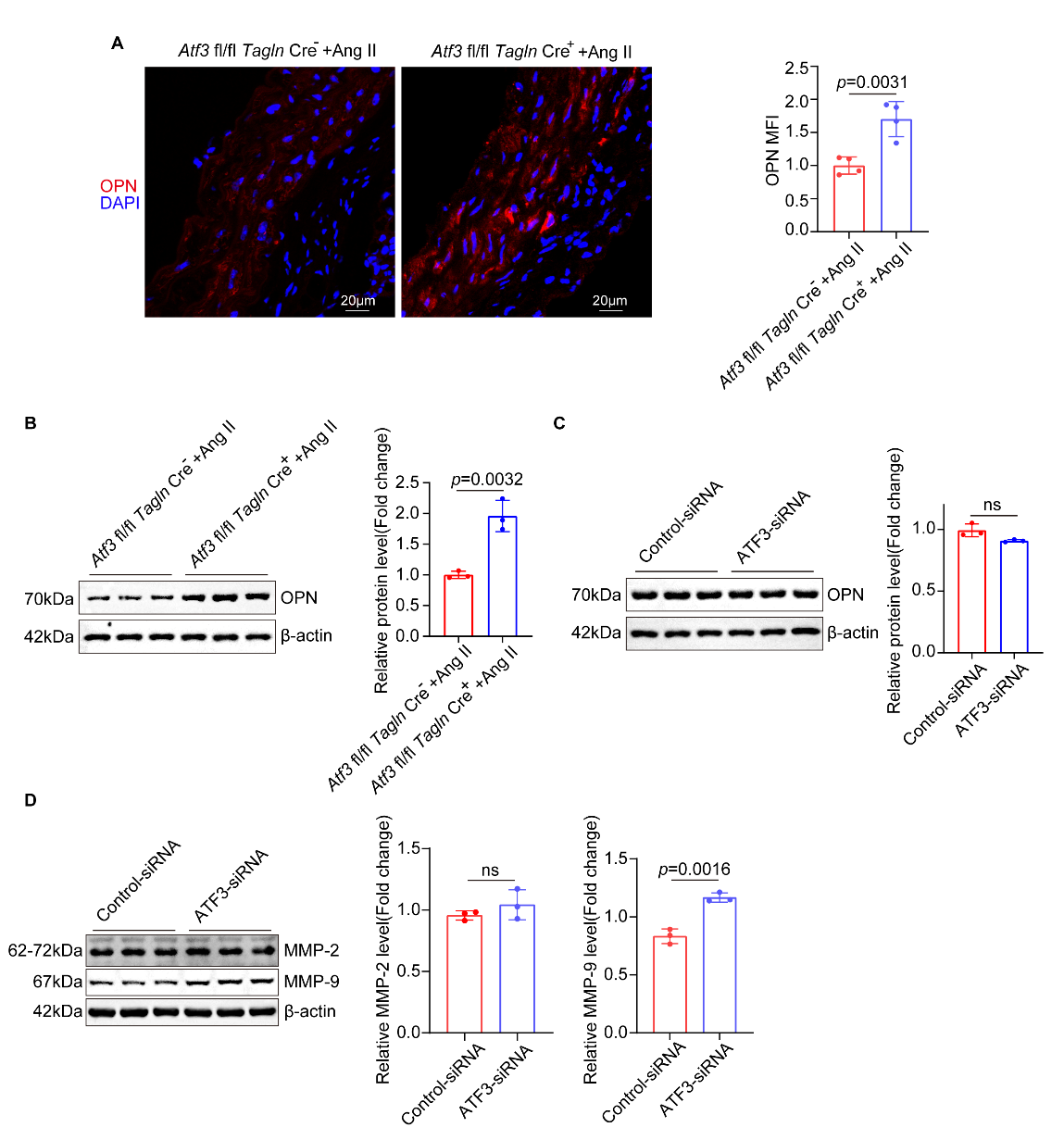


**Supplemental Figure9.**

**A**, Representative immunofluorescence staining and quantification data showing that the levels of OPN in aortic VSMCs were increased in challenged *Atf3* cKO mice compared with challenged littermate control mice (n=4 biological replicates per group, scale bar=20μm).**B**, Western blot analysis showing that the expression of OPN was increased in aortas of challenged *Atf3* cKO mice compared with challenged littermate control mice (n=3 biological replicates per group).**C**, Western blot analysis showing that the expression of OPN was no difference in H_2_O_2_ challenged VSMCs with or without ATF3 knockdown(n=3 biological replicates per group).**D**, Western blot analysis showing that the expression of MMP-9 was increased in H_2_O_2_ challenged VSMCs with ATF3 knockdown (n=3 biological replicates per group).Unpaired 2-tailed t-test was used in **A, B, C and D**


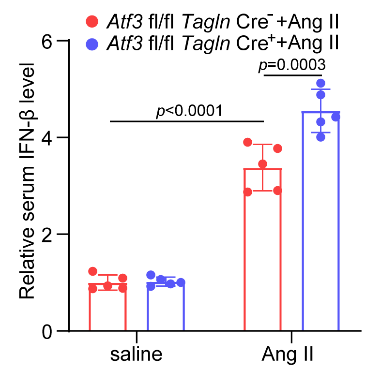


**Supplemental Figure10**

The serum IFN-β level.


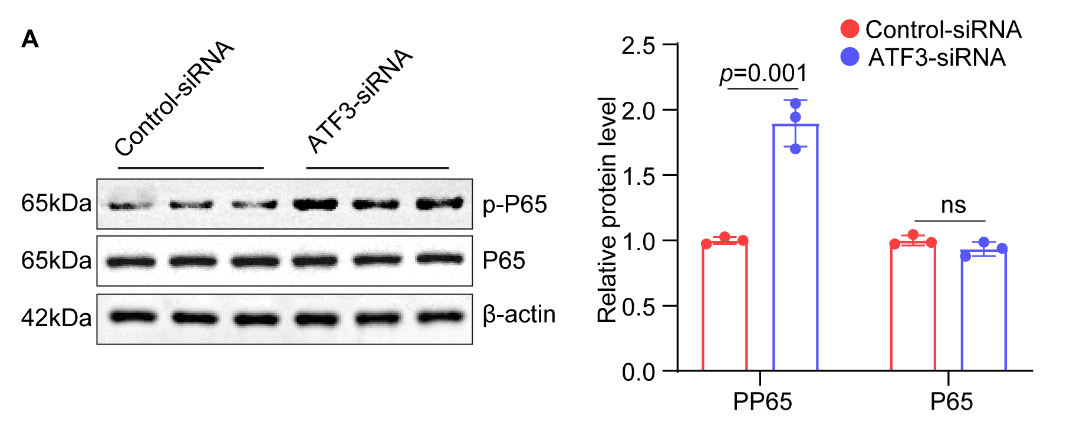


**Supplemental Figure11**

Western blot analysis showing that the expression of p-P65 was increased in H_2_O_2_ challenged VSMCs with ATF3 knockdown compared with VSMCs without ATF3 knockdown (n=3 biological replicates per group). Unpaired 2-tailed t-test was used for statistical analysis.


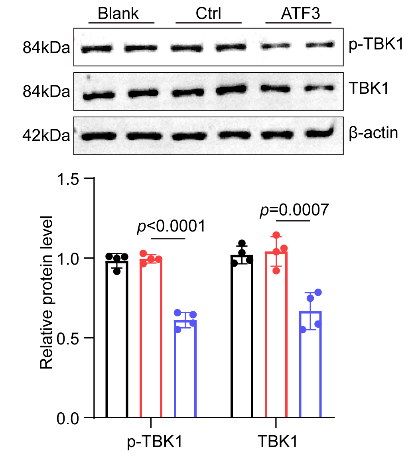


**Supplemental Figure12**

Overexpression of ATF3 in VSMCs downregulated the level of p-TBK1 and TBK1 (n=4 biological replicates per group). One-way ANOVA with the Bonferroni post-hoc test for pairwise comparisons was used for statistical analysis.


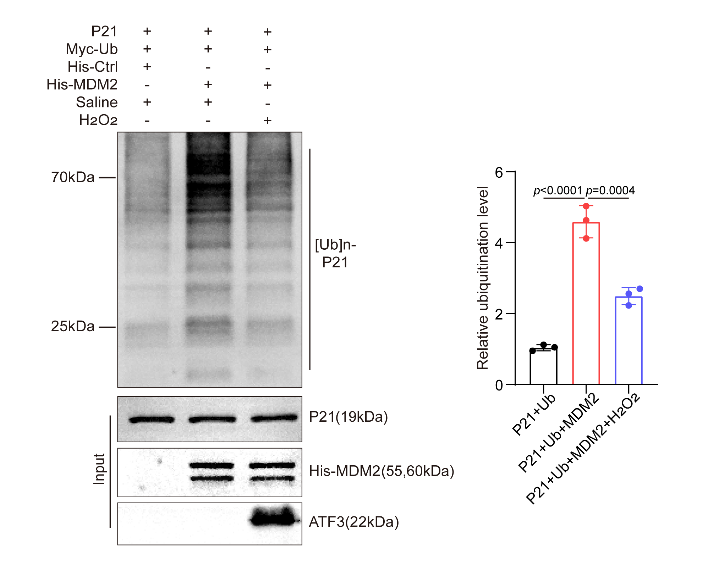


**Supplemental Figure13**

VSMCs were transfected with 0.6 μg of P21, Myc-ubiquitin, His-Ctrl/His-MDM2 for 48h and then were challenged by H2O2 for 6h. The VSMCs were collected and lysed, then were subjected to immunoprecipitation using the P21 antibody followed by SDS–PAGE. Ubiquitinated proteins were detected by western blotting using the Myc antibody. One-way ANOVA with the Bonferroni post-hoc test for pairwise comparisons was used for statistical analysis.


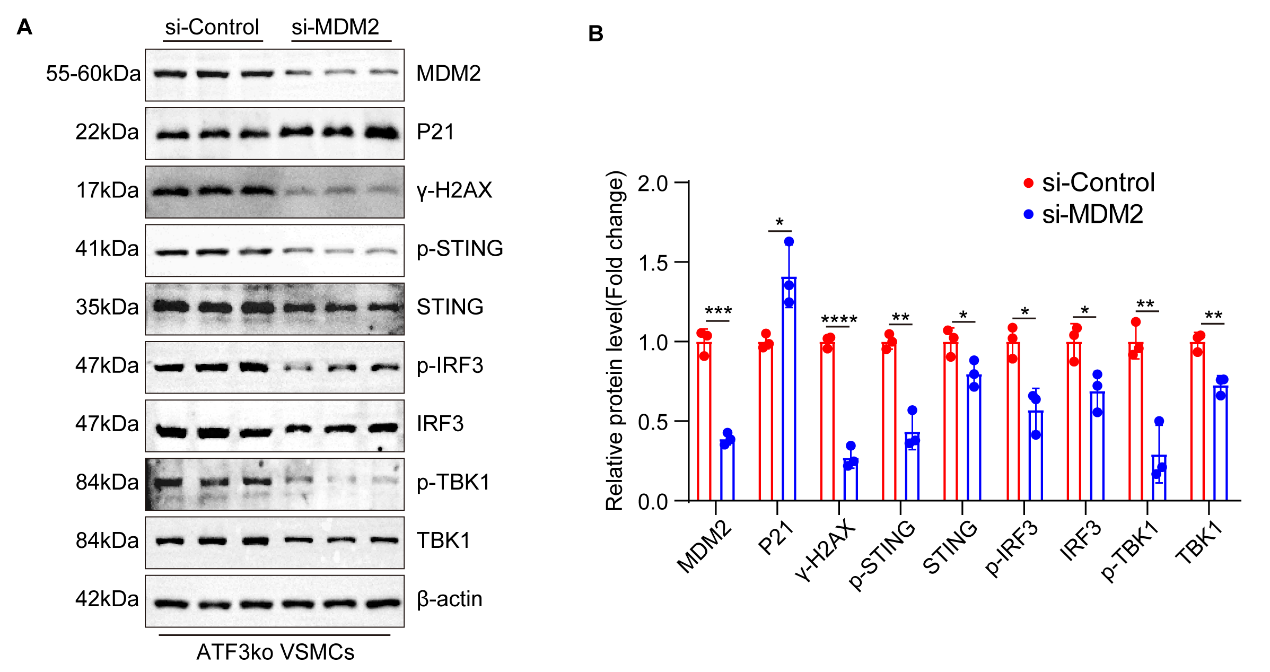


**Supplemental Figure14**

**A-B**, Western blot analysis showing that MDM2 silencing abolished the increased expression of γ-H2AX, p-STING, STING, p-IRF3, IRF3, p-TBK1 ,TBK1 and decreased expression P21 caused by ATF3 knockout in H_2_O_2_ challenged VSMCs. (n=3 biological replicates per group). Unpaired 2-tailed t-test was used in **B.**


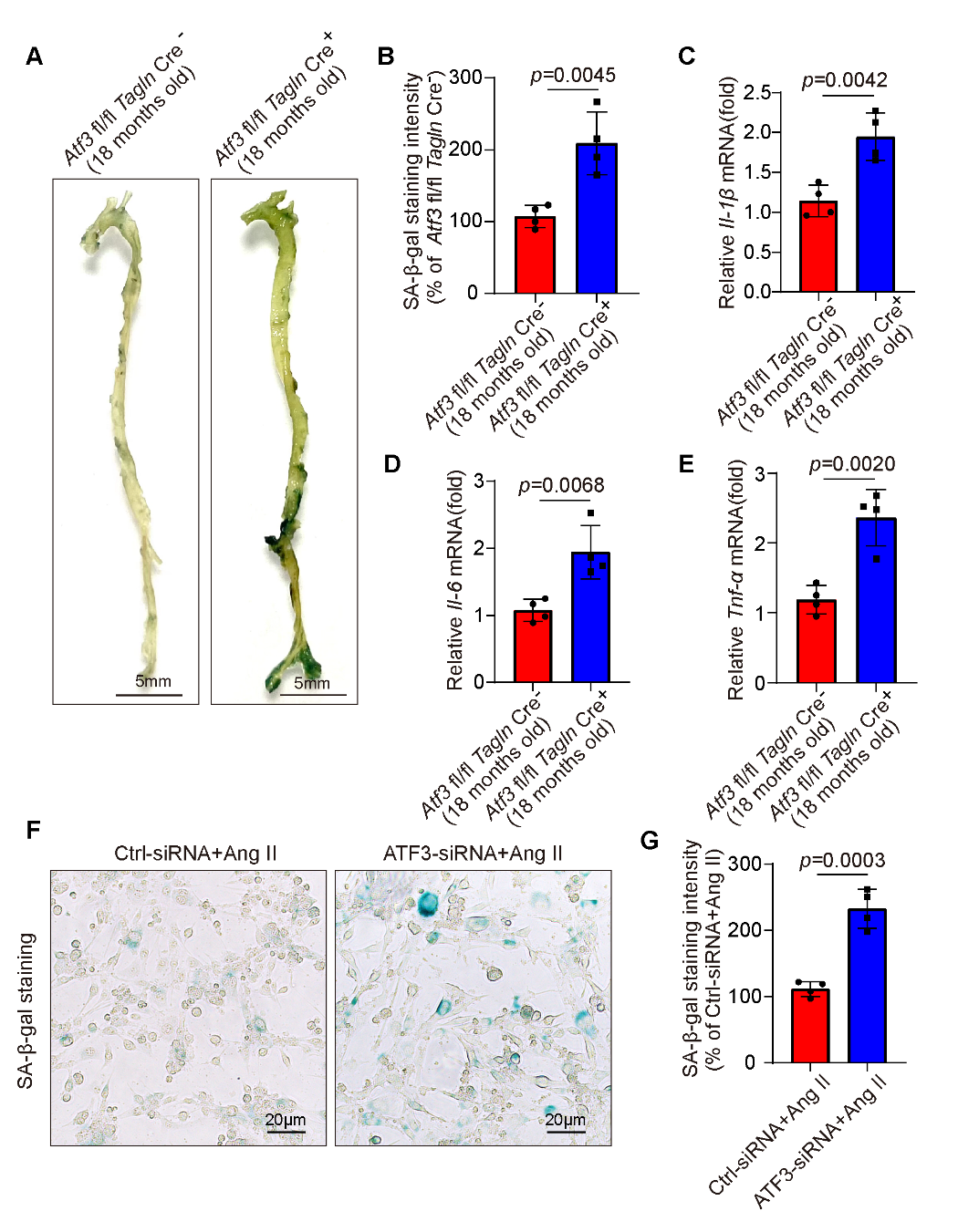


**Supplemental Figure 15. ATF3 deficiency aggravated VSMCs senescence. A** and **B**, Representative photographs and densitometric analysis of senescence-associated β-galactosidase (SA-β-gal)–staining showing that aggravated senescence in *Atf3* cKO mice compared with littermate control mice. (n=4 biological replicates per group, scale bar=5mm).**C** through **E**, Relative mRNA level of senescence-associated genes (*Il-1β*,*Il-6* and *Tnf-α*) were enhanced in *Atf3* cKO mice compared with littermate control mice (n=4 biological replicates per group). **F** and **G**, SA-β-gal staining showing that knockdown of ATF3 aggravated Ang II-induced senescence in cultured VSMCs (n=4 biological replicates per group, scale bar=20μm). Unpaired 2-tailed t-test was used in **B**-**E** and **G.**


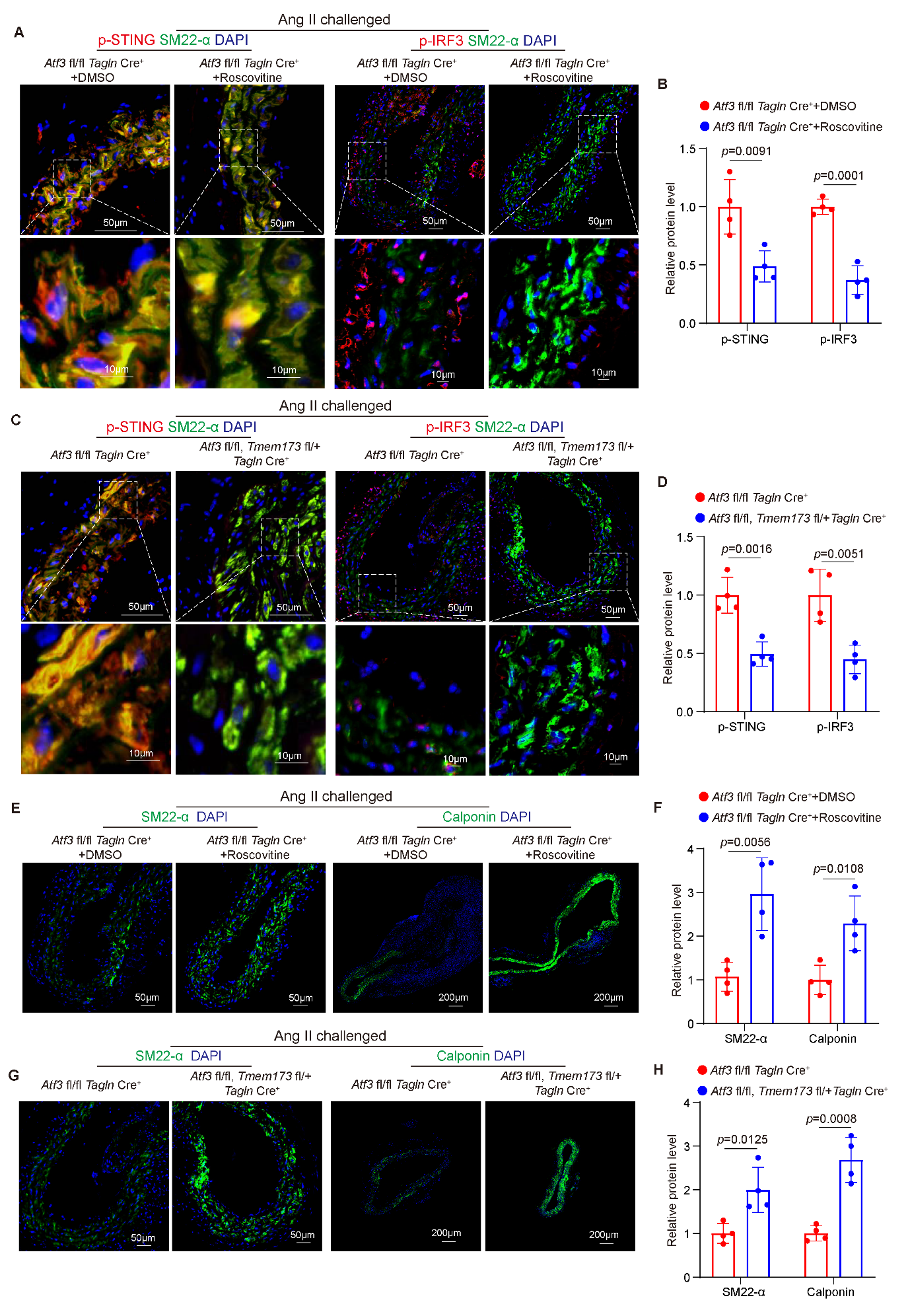


**Supplemental Figure 16.** **Roscovitine treatment or STING knockdown mitigated the adverse effects of *Atf3* knockdown on VSMCs.** **A** and **B**, The aggravated STING and IRF3 phosphorylation were reduced in the aortic wall of *Atf3* cKO mice treated with roscovitine compared with *Atf3* cKO mice treated with DMSO (n=4 biological replicates per group，scale bar=50μm in normal images, scale bar=10μm in enlarged images). **C** and **D**, The aggravated STING and IRF3 phosphorylation were reduced in the aortic wall of ATF3^fl/fl^; Tmem173^fl/+^; *Tagln*-Cre^+^ mice compared with ATF3^fl/fl^; *Tagln*-Cre^+^ mice(n=4 biological replicates per group，scale bar=50μm in normal images, scale bar=10μm in enlarged images). **E** and **F**, Decreased contractile proteins (SM22-α and Calponin) were restored in the aortic wall of *Atf3* cKO mice treated with roscovitine compared with *Atf3* cKO mice treated with DMSO(n=4 biological replicates per group，scale bar=50μm in SM22-α detection, scale bar=200μm in Calponin detection). **G** and **H**, Decreased contractile proteins (SM22-α and Calponin) were restored in the aortic wall of ATF3^fl/fl^; Tmem173^fl/+^; *Tagln*-Cre^+^ mice compared with ATF3^fl/fl^; *Tagln*-Cre^+^ mice(n=4 biological replicates per group，scale bar=50μm in SM22-α detection, scale bar=200μm in Calponin detection). Unpaired 2-tailed t-test was used in **B**, **D**, **F** and **H.**

REFERENCE:

1. Bhamidipati CM, Mehta GS, Lu G, Moehle CW, Barbery C, Dimusto PD, Laser A, Kron IL, Upchurch GJ, Ailawadi G. Development of a novel murine model of aortic aneurysms using peri-adventitial elastase. Surgery. 2012;152:238-246. doi: 10.1016/j.surg.2012.02.010
2. Voelkl J, Luong TT, Tuffaha R, Musculus K, Auer T, Lian X, Daniel C, Zickler D, Boehme B, Sacherer M, et al. SGK1 induces vascular smooth muscle cell calcification through NF-κB signaling. J Clin Invest. 2018;128:3024–3040. doi: 10.1172/JCI96477
