# Supplementary figures and images for: Deficiency of ATF3 facilitates both angiotensin II‐induced and spontaneously formed aortic aneurysm and dissection development by activating cGAS–STING pathway
Source: Clin Transl Med. 2024 Dec 27;15(1):e70147. doi: 10.1002/ctm2.70147 (PMC11680558; doi:10.1002/ctm2.70147)

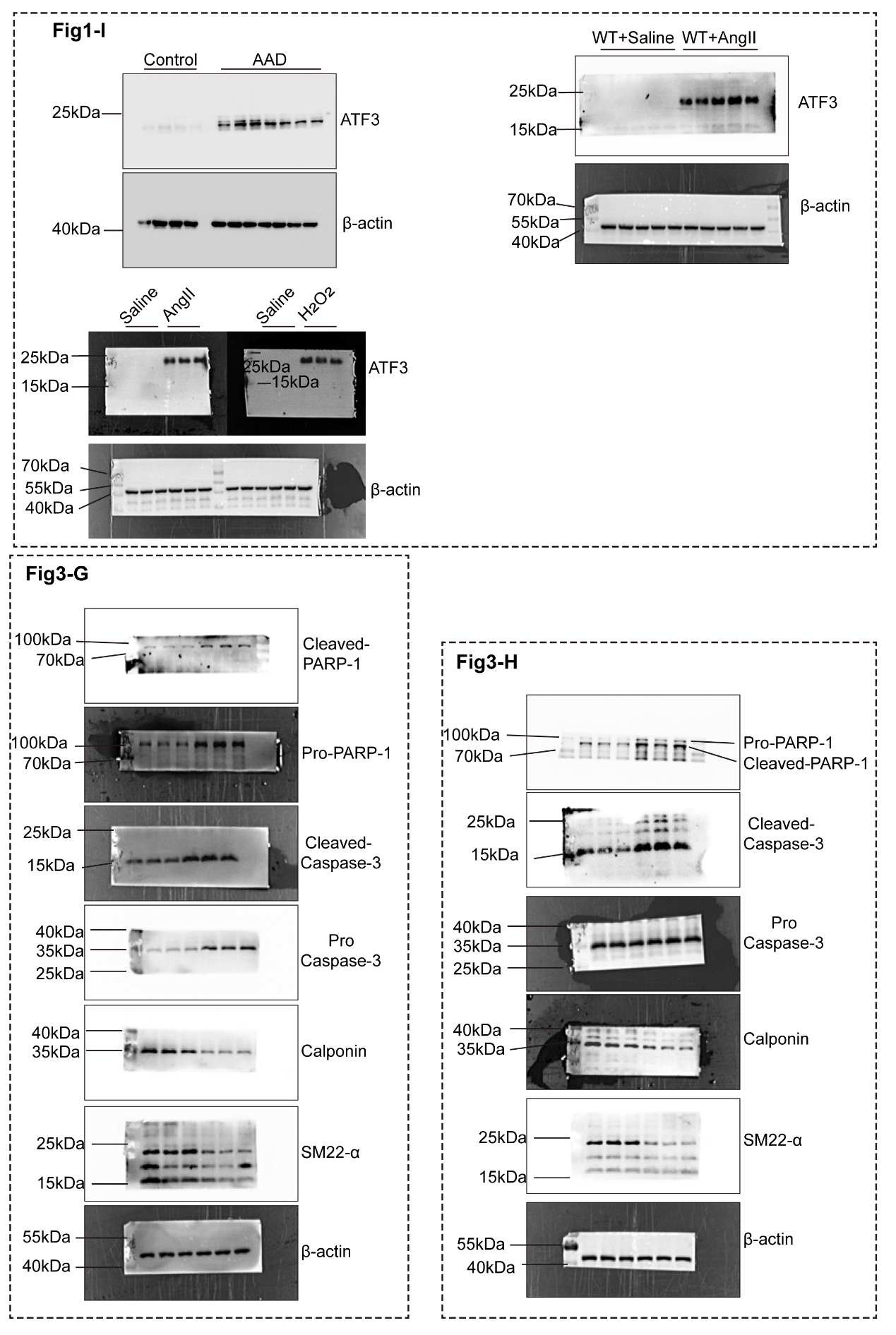

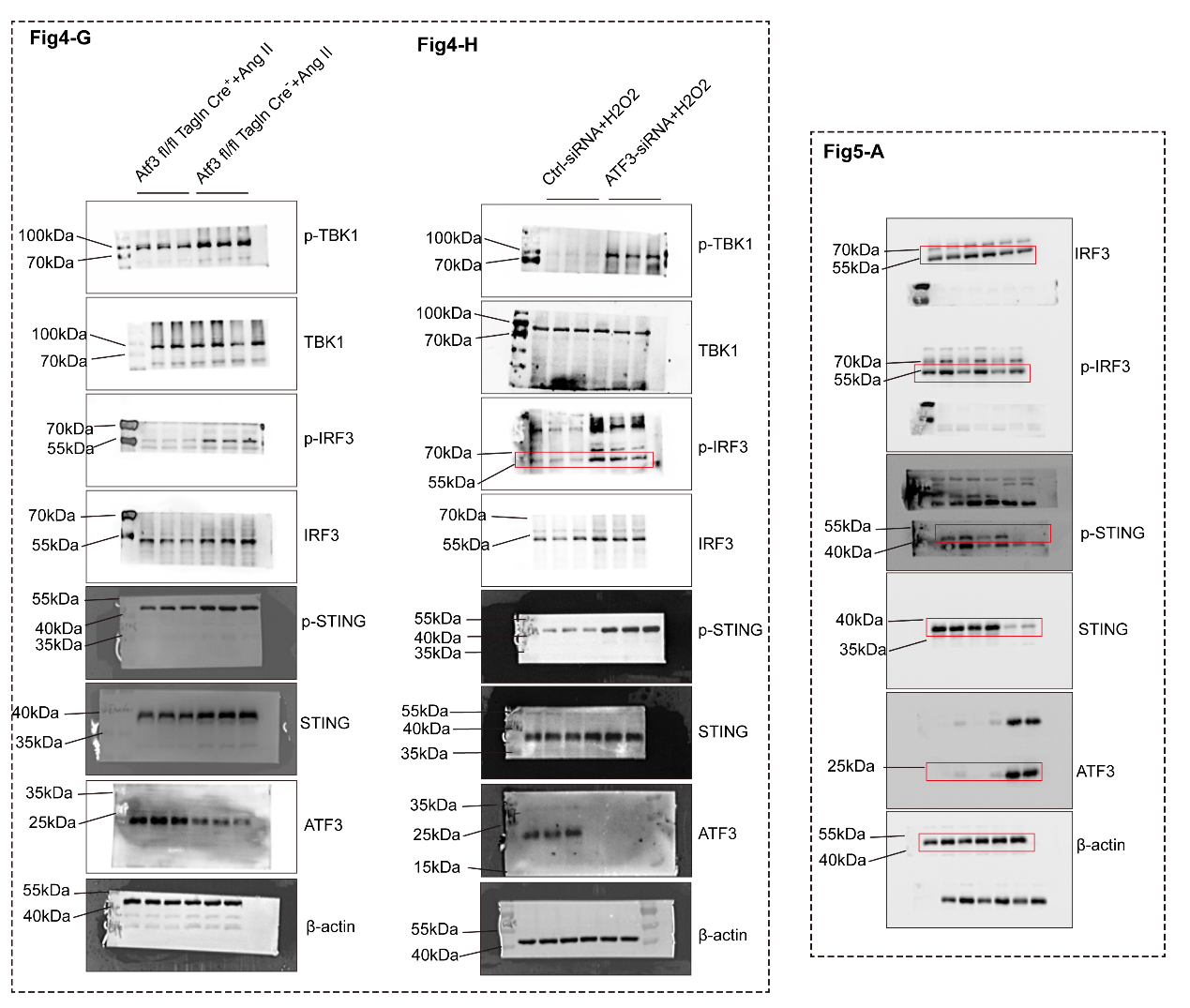

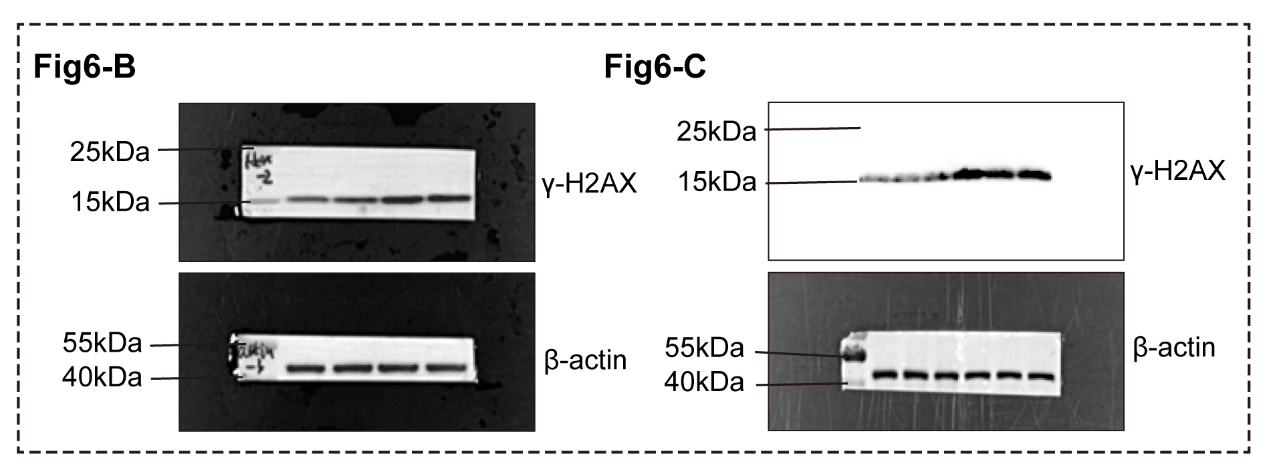

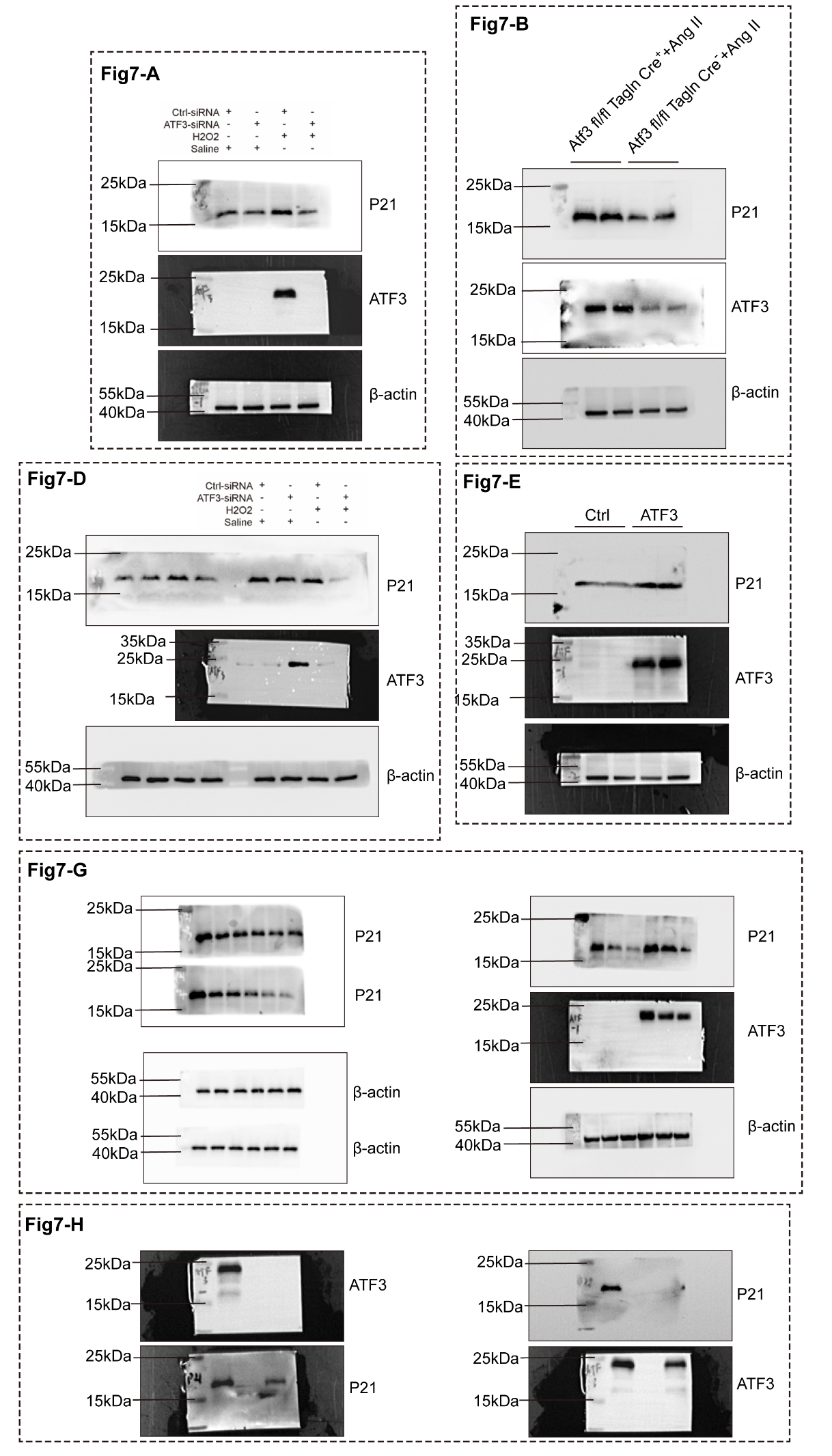

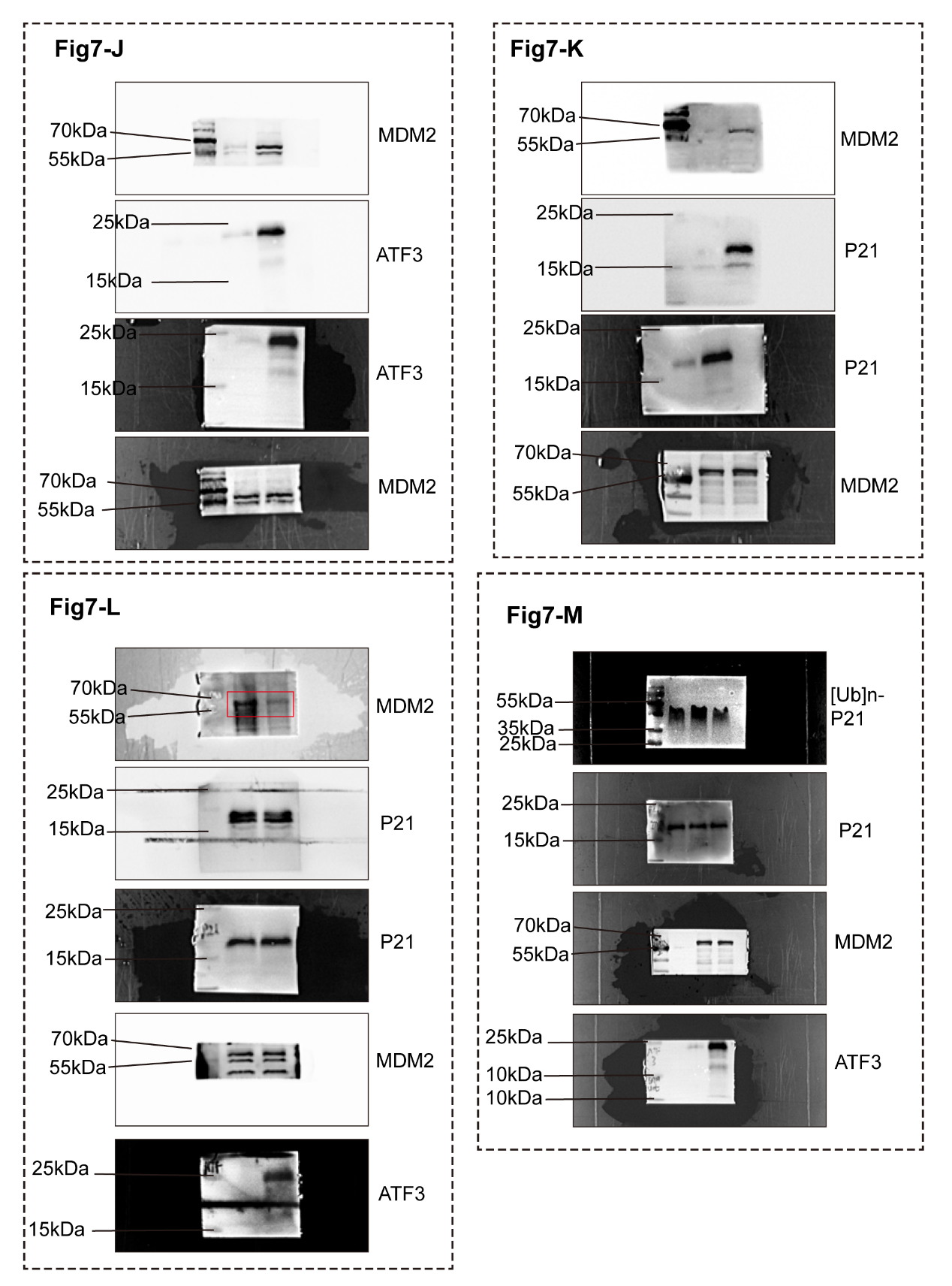

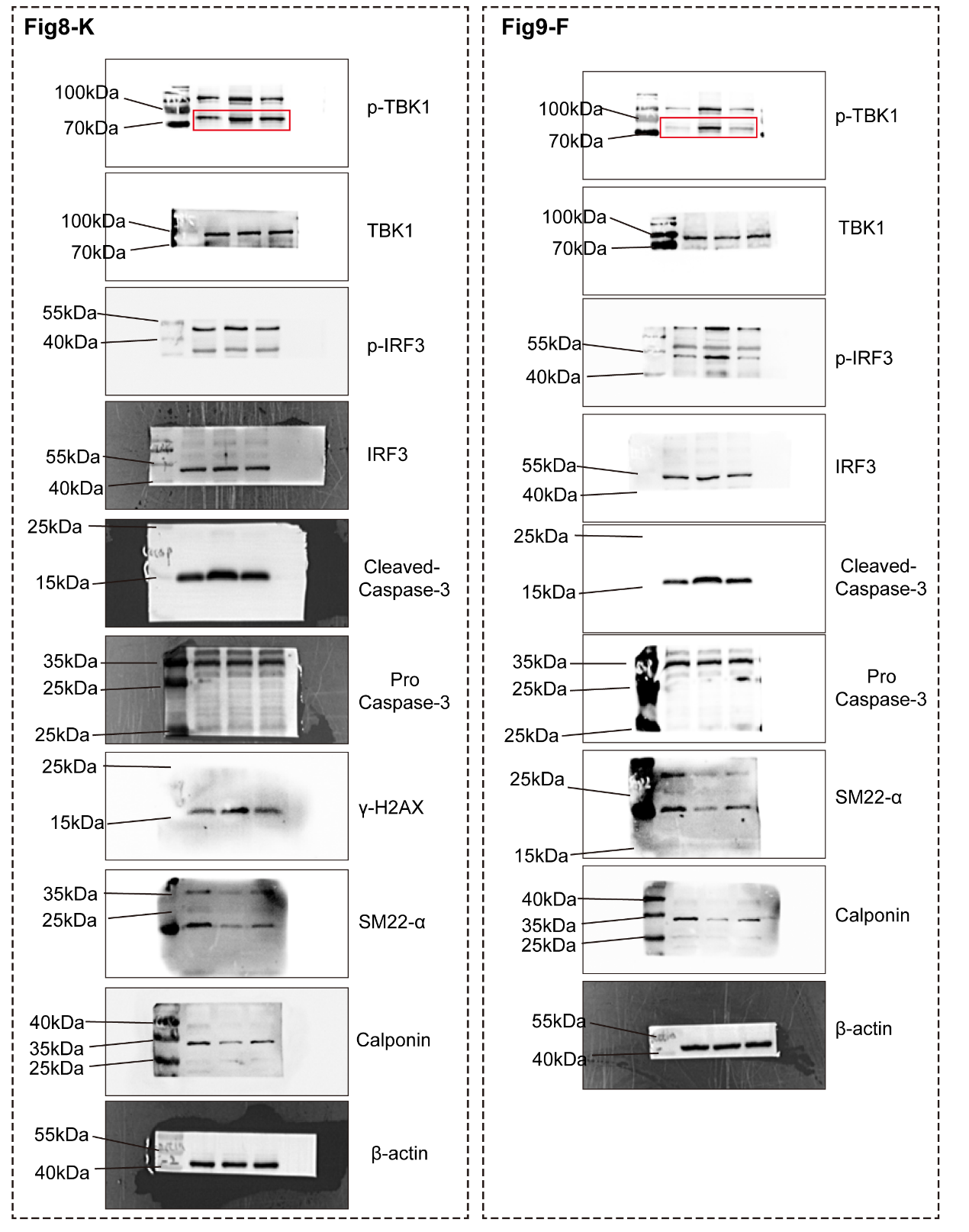

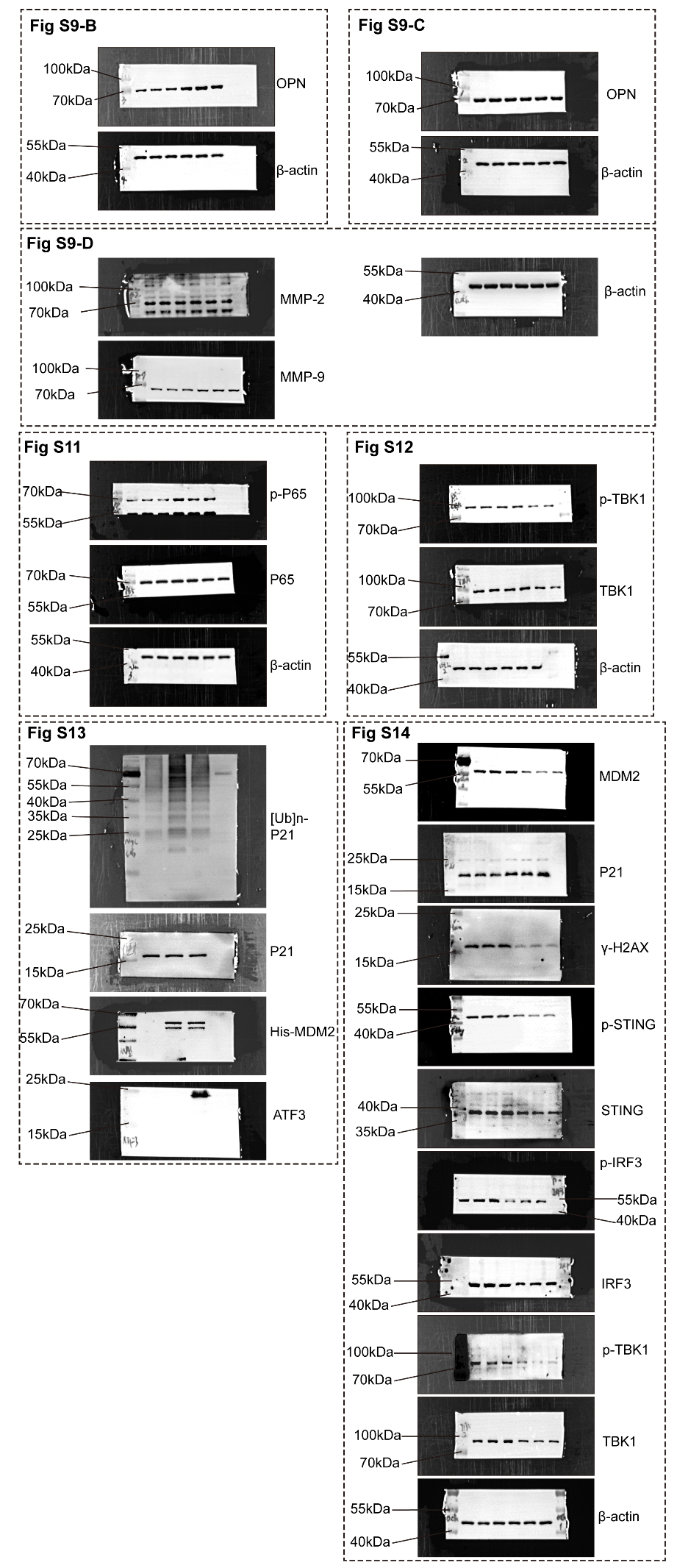

Supplement: Supplementary file 2 — Supporting Information [file CTM2-15-e70147-s001.docx]
